# Large-scale evaluation of k-fold cross-validation ensembles for uncertainty estimation

**DOI:** 10.1186/s13321-023-00709-9

**Published:** 2023-04-28

**Authors:** Thomas-Martin Dutschmann, Lennart Kinzel, Antonius ter Laak, Knut Baumann

**Affiliations:** 1 Institute of Medicinal and Pharmaceutical Chemistry, University of Technology Braunschweig, Beethovenstrasse 55, 38106 Brunswick, Germany; 2 Bayer AG, Research & Development, Pharmaceuticals, Muellerstrasse 178, 13353 Berlin, Germany

**Keywords:** Cross-validation, Deep learning, Ensemble learning, Machine learning, Uncertainty quantification, Validation

## Abstract

It is insightful to report an estimator that describes how certain a model is in a prediction, additionally to the prediction alone. For regression tasks, most approaches implement a variation of the ensemble method, apart from few exceptions. Instead of a single estimator, a group of estimators yields several predictions for an input. The uncertainty can then be quantified by measuring the disagreement between the predictions, for example by the standard deviation. In theory, ensembles should not only provide uncertainties, they also boost the predictive performance by reducing errors arising from variance. Despite the development of novel methods, they are still considered the “golden-standard” to quantify the uncertainty of regression models. Subsampling-based methods to obtain ensembles can be applied to all models, regardless whether they are related to deep learning or traditional machine learning. However, little attention has been given to the question whether the ensemble method is applicable to virtually all scenarios occurring in the field of cheminformatics. In a widespread and diversified attempt, ensembles are evaluated for 32 datasets of different sizes and modeling difficulty, ranging from physicochemical properties to biological activities. For increasing ensemble sizes with up to 200 members, the predictive performance as well as the applicability as uncertainty estimator are shown for all combinations of five modeling techniques and four molecular featurizations. Useful recommendations were derived for practitioners regarding the success and minimum size of ensembles, depending on whether predictive performance or uncertainty quantification is of more importance for the task at hand.

**Supplementary Information:**

The online version contains supplementary material available at 10.1186/s13321-023-00709-9.

## Introduction

Machine learning (ML) for drug design purposes holds a long tradition [1], but has recently started to gain further attention due to the success of deep learning (DL) [2]. Yet, the prediction of chemical properties and activities is only one step in a long and resource-intense process of drug design, discovery, and development. When developing ML models, predictions alone are not sufficient and require further analysis [3]. During model construction and testing, errors made by the model can easily be evaluated since the true target values are known. The error distribution allows the estimation of the quality of the model, but cannot be applied when predicting values for new compounds with unknown target values. In this case, it is good practice to provide an estimate of the uncertainty associated with the prediction. Measures quantifying the predictive uncertainty can be used to set a threshold which defines the model’s applicability domain. The latter is defined as follows: “The applicability domain of a (Q)SAR model is the response and chemical structure space in which the model makes predictions with a given reliability.” [4]. The reliability of a model can either be addressed by quantifying its confidence, or, conversely, its uncertainty. Recent studies make use of the term uncertainty quantification (UQ) [5]. Uncertainty can be of aleatoric nature, relating to the random process that generates the target values, or epistemic nature, implying model-related uncertainty [6]. Usually, these two types cannot be fully distinguished [7].

Classification algorithms often provide built-in mechanisms or augmentations to measure their uncertainty [8]. One example is estimating posterior probabilities (through Platt scaling [9]) when using support vector machine (SVM) classifiers [10]. When using SVMs for regression [11], however, no such UQ measure exists. In cases where the model provides no built-in uncertainty quantifier, practitioners use generic methods that are model-independent. A common procedure in such a situation is to compute ensembles of models instead of a single estimator [12]. When an ensemble predicts the output of a given instance, it is presented to each individual model, which makes a single-valued prediction. These predictions are then combined to a single output by averaging them. Let $\hat{y}_{i}^{test}$ denote the prediction for a given instance from the test set, computed by ensemble member *i* (out of *M* members). Then, the final prediction that the ensemble reports for that instance is obtained as follows:

1 $$\begin{aligned} \begin{aligned} \hat{\bar{y}}^{test} = \frac{1}{M} \sum _{i=1}^M \hat{y}_i^{test} \end{aligned} \end{aligned}$$

In the case of this study, the different ensemble members are generated by subsampling. Initially, ensembles were suggested to improve the predictive performance: Errors as a result of model variation are reduced since averaging across models smooths out those variances. This procedure also inherently yields a measure for UQ: The variability between the individual predictions for a specific compound quantifies the uncertainty of the whole ensemble for that instance. Regression tasks typically compute the average of the predictions as a point estimator and the standard deviation or variance as UQ measure [13–15]. In case of the standard deviation, the uncertainty $\hat{u}$ of $\hat{\bar{y}}^{test}$ is then computed as follows:

2 $$\begin{aligned} \begin{aligned} \hat{u} = \sqrt{\frac{1}{M} \sum _{i=1}^M \left( \hat{y}_i^{test} - \hat{\bar{y}}^{test}\right) ^2} \end{aligned} \end{aligned}$$

In terms of uncertainty analysis, this procedure estimates the epistemic uncertainty [16].

Random forests (RFs) [17], consisting of individual decision trees, are already ensembles by design, hence the ensemble variance is their default UQ measure. Although it is possible to conceive alternative methods when using RFs, the default approach seems to prevail [18]. In DL, the dropout technique originating from regularization has been successfully transferred to generate UQ estimates [19], also known as Monte Carlo (MC) dropout [20]. For a fitted neural network, a fixed fraction of randomly selected weights is deactivated during inference. Now, the output of a single instance can be predicted multiple times using a single network but different deactivated weights. Basically, UQ by MC dropout simulates an ensemble since each prediction per dropout mask can be considered to come from a different model [21]. With the focus shifting more and more towards DL, novel UQ measures are developed that are specifically designed for neural networks [22–25]. A very promising approach has been introduced by Soleimany et al. [26]. Their application of evidential DL for cheminformatic regression tasks appears more efficient compared to ensembles and MC dropout. Nonetheless, depending on the dataset at hand, the success of ensembles can vary with the number of ensemble members and the strategy to generate ensembles [27]. The type of molecular featurization can influence the outcome as well [28]. The number of possible object representations to choose from is large in cheminformatics [29]: Among them, there are structural descriptors, physicochemical descriptors [30], and certain descriptors generated by deep neural networks (DNNs) from structural inputs such as SMILES or molecular graphs. The latter descriptors comprise latent-space encodings [31] and recent graph convolutional neural networks, where the model automatically learns the optimal weighting of each subgraph contribution [32]. The combination of graph-based learned representations with DNNs is capable of surpassing traditional approaches [33]. Unfortunately, graphs cannot be applied to ML techniques that strictly require structured data, such as RF or SVM. Based on SMILES-to-SMILES autoencoders, task-independent latent-space encodings provide learned molecular representations that can be used as inputs for traditional ML algorithms. One example that is publicly available and applicable for ML related to drug discovery is continuous and data-driven descriptor (CDDD) developed by Winter et al. [34].

In theory, the ensemble method should provide UQ estimates for all settings. Although it might be outperformed at some point, it is expected to yield reasonable uncertainty estimates. UQ based on ensembles can also be used to calibrate conformal predictors [35]. However, ensembles also come with method-specific disadvantages. Obviously, an ensemble requires longer training and inference times and more disk space than a single model. For most practitioners, ensembles are therefore inconvenient to deploy, although sometimes no alternative is at hand.

To gain a better insight on the applicability of ensembles for UQ, a diverse collection of datasets has been compiled, including various tasks. Using four molecular featurizations, from traditional fingerprints to (task-unspecific) learned representations, the usefulness of the ensemble method is evaluated for neural networks and three other well-established supervised ML techniques. The dependency on the number of ensemble members (the ensemble size) and the comparison to model-specific measures (if available) is outlined. Prior studies also deal with the evaluation of different UQ methods and ensembles, but with less datasets and different focuses [14, 36], e.g., exclusively for DNN ensembles of message passing neural networks [37].

## Results and discussion

### Predictive performance

**Fig. 1:**
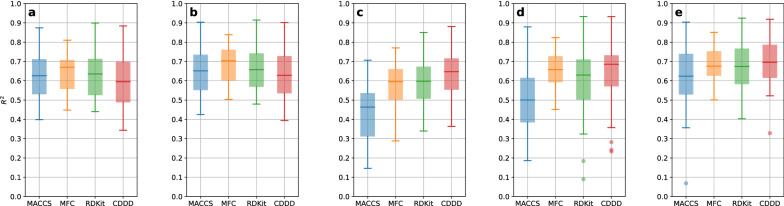
Overview of all predictive performances ($R^2$) for the modeling techniques RF (**a**), XGB (**b**), SVM (**c**), SNN (**d**), and DNN (**e**) as box-whisker plots. The featurization-specific results (for MACCS, MFC, RDKit descriptors, and CDDD) are shown and colored separately within each plot. The only negative $R^2$, corresponding to MMP2, using RDKit descriptors and the SNN, was omitted for visual clarification

Subsampling ensembles of 200 members were generated by running 200 2-fold cross-validations (CVs) for all datasets, in combination with all molecular featurizations, and all modeling techniques, resulting in 640 combinations (32 datasets * four featurizations * five modeling techniques = 640). The final prediction of the ensemble for each compound was obtained by averaging over the 200 individual member predictions. It can be seen in Fig. 1 that predictive performances are on average comparable across featurizations and modeling techniques, with MACCS [38] showing the largest variability in performance. Morgan fingerprint count (MFC) shows the smallest performance variability. Either MFC or CDDD achieved the highest average performance for each modeling technique. An exhaustive overview for all individual predictive performances of each of the full 200-member ensembles obtained by 2-fold CV is provided in Additional file 1: Fig. S1.

**Fig. 2:**
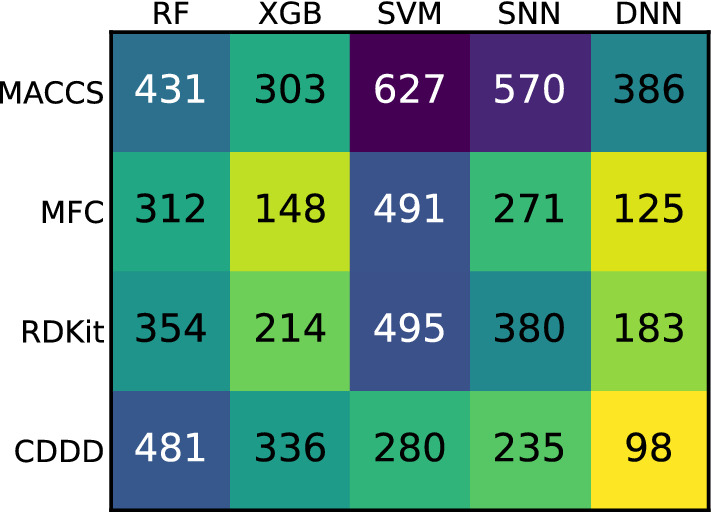
Rank sums for each combination of featurization and modeling technique across all datasets, summed up for predictive performance. Smaller is better, as the rank for each combination (one to 20) was summed up for all datasets. The best performance within a dataset was assigned rank one and the worst rank 20. Cells containing smaller values are colored brighter

Although the box plots capture the variability between the modeling techniques, certain trends are hardly perceptible but can be visualized using rank sums. The sum of ranks for each combination of featurization and modeling technique across all datasets are shown in Fig. 2. Here, it can be seen that the four highest ranking (i.e., best) combinations were DNNs together with MFC, RDKit descriptors [39], and CDDD, and XGBoost (XGB) [40] together with MFC. Out of the four lowest ranking (i.e., worst) combinations, three involved SVMs, namely combined with MACCS, MFC, and RDKit descriptors. The shallow neural network (SNN) in combination with MACCS achieved the second lowest rank sum. Combinations involving MACCS in general turned out to be underperforming when compared to other featurizations.

Several factors were kept constant in this benchmark. Varying the random seed, the number of folds, choosing a different set of fixed hyperparameters, or hyperparameter tuning would expectedly lead to minor performance differences on the level of individual models. Besides several alternative *k*s for *k*-fold CV, subsampling ensembles of 200 bootstrap samples were also tested for some of the datasets, but were found to perform similarly. See Section 2 in Additional file 1 for a discussion of the comparison between the two subsampling methods.

For neural network ensembles, the predictive performances fluctuated heavily within a dataset between featurizations. Although ensembles employing DNNs were closely the most successful modeling technique, they also accounted for some of the worst predictive performances in the whole study at the same time, e.g., $R^2=0.0$ $7$ for dataset MMP2 [41], featurized by MACCS. Interestingly, MACCS was the only featurization that did not perform well in combination with the DNN. In contrast to traditional ML methods, the hyperparameter choices for DL are far more complex. E.g., the network architecture alone offers a huge number of reasonable settings, while most traditional ML techniques rely on a small set of hyperparameters. Generalizing settings for DNNs remains an active field of research [42]. As “universal approximators” [43], they have the capability to fit any function given sufficient data and proper hyperparameter settings. As a consequence, generic “off-the-self” neural networks are not as robust as traditional ML techniques which bears the risk that they are not suited for a specific dataset/featurization combination. Some of RDKit’s descriptor values get extraordinarily large for a small number of molecules in the dataset [33], which causes a wide variation of neural network performance. If all of these extreme outliers happen to be part of the test split, a model might produce highly erroneous predictions for them. These predictions will be within the training output range when using RFs and RF ensembles since they are unable to extrapolate, which does not hold for DNNs. To deal with such extreme outliers, predictions outside two times the training output range were eliminated. A discussion about the issue of removing prediction outliers is provided in Section 3 in Additional file 1.

Ensembles of the tree-based methods RF and XGB predicted with rather constant performance. Single XGB models on default settings without any additional regularization might be overfitted, which is why they could benefit from being members of an ensemble. The steep rise in cumulative $R^2$-values for several XGB models when accumulating more and more members confirms this assumption (data not shown here, but visualized for all datasets and combinations in [44]). The success of the combination of XGB and MFC is particularly noteworthy because MFC is never the most performant featurization in the other combinations. Conversely, tree-based methods were the only modeling techniques which did not result in the smallest (i.e., best) rank sums when combined with CDDD.

Performances of SVM ensembles were mostly located below those of neural networks and tree-based techniques. In combination with MACCS, SVM ensembles showed the lowest performance, followed by MFC. To explain in detail why specific combinations do not lead to promising results, or why some modeling techniques lead to exceptionally good results for usually unfavorable featurizations, is not within the scope of this study. Nonetheless, other studies investigating performance differences noticed similar relationships. One example is the performance difference between MACCS and featurizations based on extended-connectivities (albeit fingerprints and not counts) [45, 46].

**Fig. 3:**
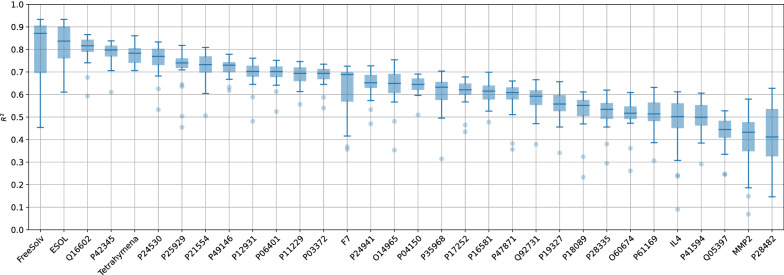
Overview of all 200-member ensemble predictive performances ($R^2$) for each dataset as box-whisker plots, sorted by descending median. The only negative $R^2$, corresponding to MMP2, using RDKit descriptors and the SNN, was omitted for visual clarification

Some datasets seem to be easier to model than others, e.g., Tetrahymena [47] constantly achieved an $R^2$ of 0.71 or higher. On the other hand, some datasets appear to be particularly hard to model. No predictive performance above 0.62 was achieved for MMP2, IL4, O60674, P18089, P28335, P28482, P41594, P61169, and Q05397, regardless of the setting. The success was therefore less dependent on the chosen combination but more on the dataset. Figure 3 illustrates this observation. While intra-dataset variability is typically small, inter-dataset variability spans a large range.

In general, the three datasets not related to bio-activity seemed to be relatively easy to model. As already pointed out in another study from this group [18], the endpoints of FreeSolv are based on simulations and were therefore computed instead of measured [48]. This might explain why modeling FreeSolv usually turns out successfully. Bio-activity datasets were, on average, harder to model. For some of them, e.g., Q05397, the dependent values did not seem to be normally distributed, and many of them (roughly 18%) were also censored. The occurrence and severeness of activity cliffs adds to the modeling difficulty in general [49], regardless of using ensembles or single models.

**Fig. 4:**
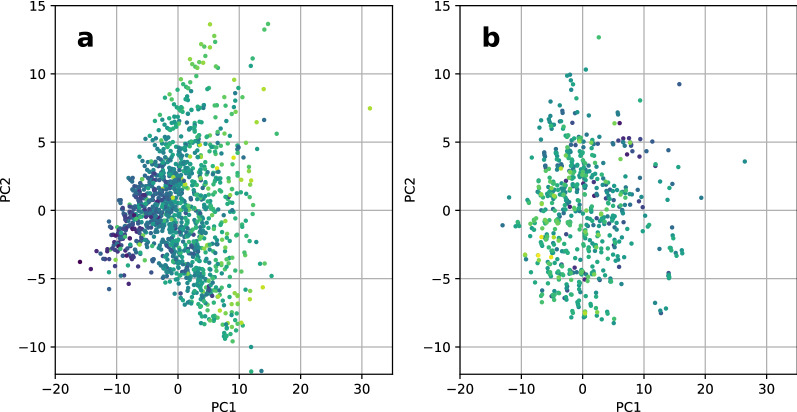
Scatter plot of the first two components after applying principle component analysis to RDKit descriptors, colored according to the corresponding output value (brighter is larger), for Tetrahymena (**a**) and IL4 (**b**)

To support these explanations, patterns in the data were visualized by performing dimensionality reduction followed by coloring each embedded compound according to its target value (darker for lower values, brighter for higher values). Tetrahymena and IL4, featurized using RDKit descriptors, exhibit different output distributions when plotting the first two components after applying principal component analysis, as visualized in Fig. 4. While the plot for Tetrahymena shows a color gradient, the plot for IL4 shows no gradient or areas that appear homogeneous in color distribution. This suggests that the relation between inputs and outputs could be easier to learn for Tetrahymena than for IL4.

### Uncertainty quantification performance

The standard deviation of the 200 predictions made by the ensemble members was applied to quantify the uncertainty of the average prediction (i.e., the output of the ensemble). UQ performance was assigned using Spearman’s rank correlation coefficient ($\rho$), measuring how well UQ values reflect the magnitude in error (cf. Quality assessment in the Materials and methods section). Similar to $R^2$, larger values of $\rho$ are desired, as they indicate a better ability of the UQ measure to rank the predictions according to their absolute prediction error. All individual $\rho$-values can be found in Additional file 1: Fig. S4.

**Fig. 5:**
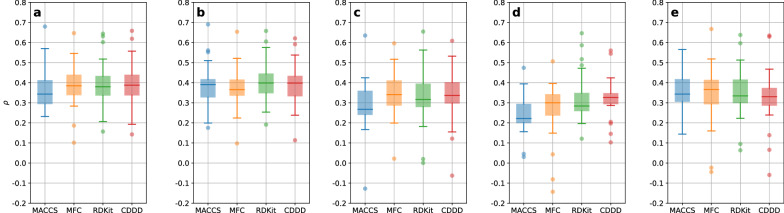
Overview of all UQ performances ($\rho$) for the modeling techniques RF (**a**), XGB (**b**), SVM (**c**), SNN (**d**), and DNN (**e**) as box-whisker plots. The featurization-specific results (for MACCS, MFC, RDKit descriptors, and CDDD) are shown and colored separately within each plot

As visualized in Fig. 5, UQ performances across featurizations and modeling techniques were comparable on average, although values of $\rho$ appear more scattered than $R^2$ values, as indicated by more outliers outside the range of the whiskers.

**Fig. 6:**
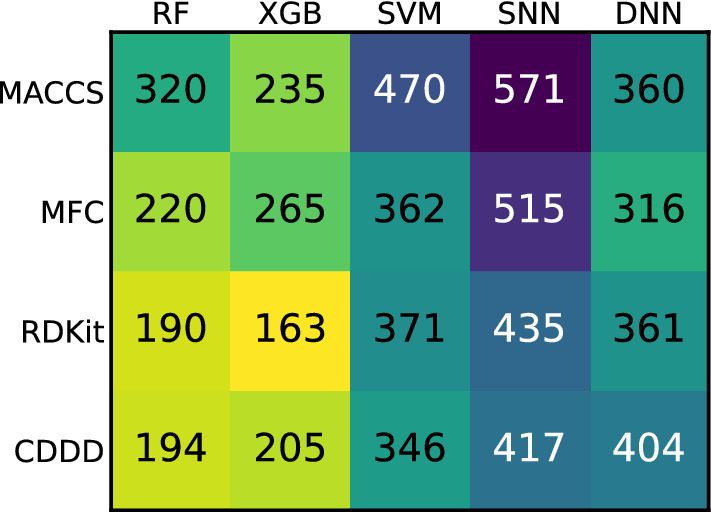
Rank sums for each combination of featurization and modeling technique across all datasets, summed up for UQ performance. Smaller is better, as the rank for each combination (one to 20) was summed up for all datasets. The best performance within a dataset was assigned rank one and the worst rank 20. Cells containing smaller values are colored brighter

Again, the influence of the combination on the success of UQ was clarified using the sum of ranks, which are shown in Fig. 6. As can be seen from the rank sums, combinations involving MACCS mostly underperformed in UQ, comparable to their relatively low predictive performance. The modeling technique with the lowest UQ performance was constantly SNN. In general, the difference in rank sums between lowest and highest performing combinations was lower for UQ performance than for predictive performances.

Ensembles of RFs, like XGB ensembles, did not only exhibit less variability for predictive performance, they were also more successful in quantifying the uncertainty than ensembles of most other techniques. Since the ensemble member disagreement is already the established uncertainty measure for RFs, it was expected that the corresponding uncertainty of ensembles of RFs will perform similarly. Overall, UQ performances of RF and XGB were better when using the two continuous featurizations RDKit descriptors and CDDD, instead of count-based featurizations.

**Fig. 7:**
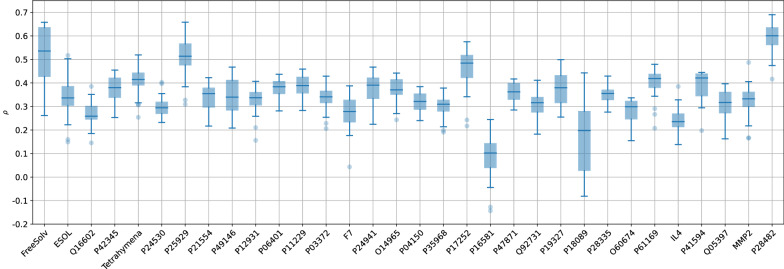
Overview of all 200-member ensemble UQ performances ($\rho$) for each dataset as box-whisker plots, in the same order as in Fig. 3. The absence of a trend (as compared to Fig. 3) shows the lack of the correlation between median $R^2$ and median $\rho$ (see also Fig. 8)

The comparison of the performance of UQ summarized in Fig. 7 shows again the large influence of the dataset, rather than of featurization or modeling technique.

Tetrahymena is an example for a dataset whose models exhibited high predictive performances and high UQ performances alike. From the theoretical point of view, the ensembles therefore have relatively small prediction errors, i.e., the average of most ensemble predictions is close to the observed value. Moreover, in cases where the mean prediction is further away from the given output, there is also more variation in the vector of predictions. An example for a dataset that appears easy to model despite the fact that its uncertainties do not seem to reflect the magnitude of the prediction errors is Q16602. In theory, there are two possible underlying scenarios that account for low UQ performance despite reasonable predictive performance: First, although most mean predictions are reasonable estimations, the standard deviations could be comparable for all predictions, therefore making a distinction between certain and uncertain estimations impossible. Second, the phenomenon occurs when too many uncertainty values are misleading (i.e., low uncertainties for large errors and high uncertainties for small errors). P28482 is considerably hard to model, yet the corresponding $\rho$-values are among the highest in this study, making it the counterpart of Q16602. Here, larger standard deviations indicate more erroneous predictions, although these predictions are rather bad. Modeling IL4 results in low predictive performances as well as in low UQ performances. For very badly performing models yielding predictions that almost appear to be random, none of the two performance qualities are expected to be high.

**Fig. 8:**
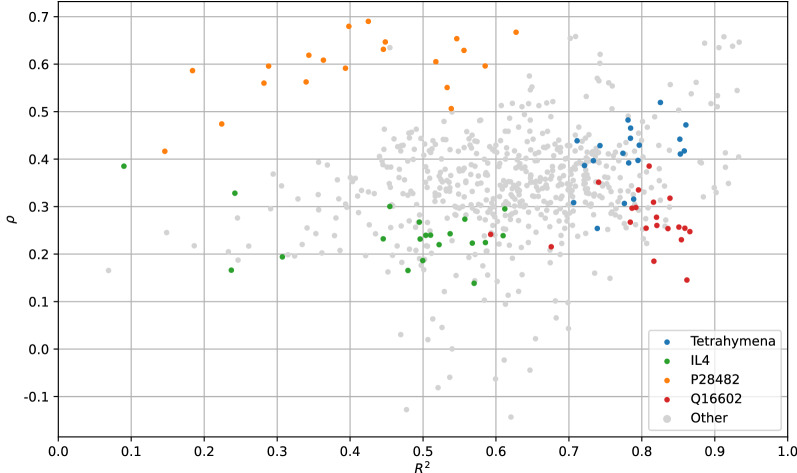
Predictive performances ($R^2$) vs. UQ performances ($\rho$) for all 200-member 2-fold CV ensembles. The outcomes for some datasets are colored according to the dataset

The results indicate that for the ensemble method, there is no connection between predictive performance and UQ performance, i.e., better ensembles are not necessarily accompanied by better UQ performance, nor worse UQ performance. The lack of correlation between predictive performance and UQ performance is further highlighted by plotting the $R^2$-values against the $\rho$-values, visualized in Fig. 8. Here, several examples of datasets are highlighted that predominantly populate corners of the plot.

### Determining the smallest ensemble size for meaningful performance improvement

Both predictive performance and UQ performance are expected to improve when increasing the number of ensemble members. To validate whether this expectation holds, the performance was computed for growing ensembles, i.e., computing the performance of ensemble size one (single models), then of ensemble size two, ensemble size three, and so on. For UQ performance, the minimum number of members started at two, as at least two predictions are required to compute the standard deviation.

**Fig. 9:**
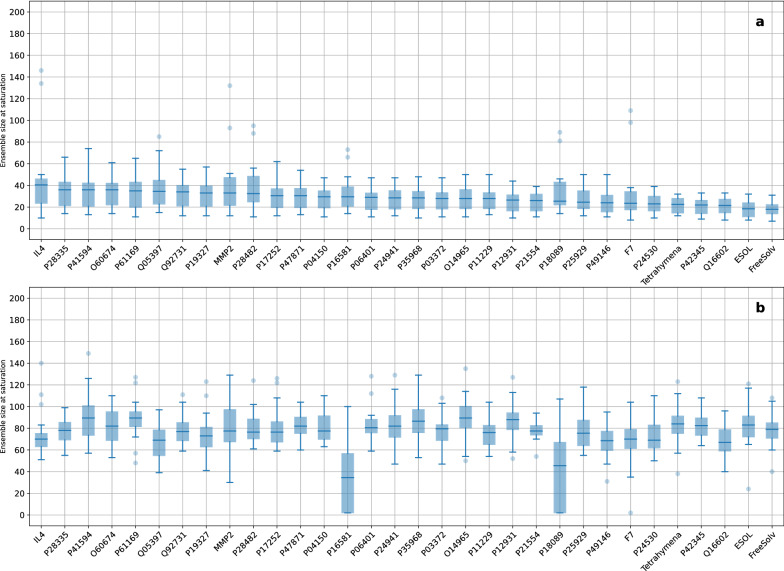
Overview of the ensemble sizes where the saturation was reached for each dataset as box-whisker plots, for predictive performance (**a**) and UQ performance (**b**). The box-whisker plots are sorted by descending median of the predictive performance

To reduce chance effects owing to the order in which the members were originally generated, cumulative member curves were computed by randomly permuting the original member order 200 times for each setting, followed by taking the median curve. Saturation functions were fitted through the median curves to estimate when the cumulative member curves have reached their plateau and the ensemble would not benefit from additional members. A detailed example of the curve processing steps is provided in Section 5 in Additional file 1. The distributions of the ensemble sizes where the saturation was reached are shown in Fig. 9.

It can be seen in Fig. 9 that improving UQ performance to the point of estimated saturation requires substantially more members than improving predictive performance. This is reflected in the median ensemble size at saturation, which ranges from 18 to 40.5 for predictive performance, and from 34.5 to 89.5 for UQ performance. The five datasets that required the smallest ensemble size to reach saturation for predictive performance (Tetrahymena, P42345, Q16602, ESOL, and FreeSolv) are also those for which the highest median $R^2$ values were achieved for the full ensembles (see Fig. 3). Furthermore, the number of required ensemble members at saturation appears to be anti-correlated to the predictive performance of the full ensemble (see Additional file 1: Fig. S6). An explanation could be that these datasets are already easy to model, so more members do not provide as much gain as for datasets that are harder to model. Figure 9 also shows that there is again no connection between predictive performance and UQ performance, i.e., datasets that require fewer members to reach the saturation for predictive performance do not necessarily also require fewer members to improve UQ performance.

**Fig. 10:**
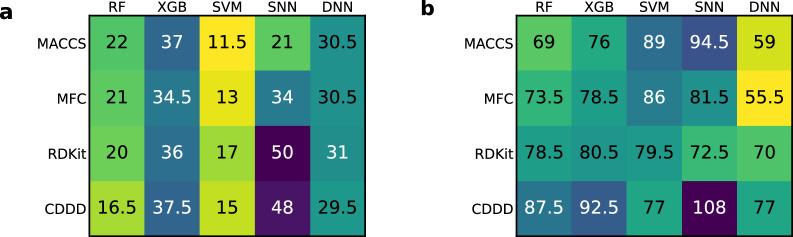
Median ensemble sizes where the saturation was reached across all datasets for each combination of featurization and modeling technique, for predictive performance (**a**) and UQ performance (**b**). The cells are colored according to their values (smaller is brighter)

To check whether the featurization or the modeling technique are influencing the rate of performance gain, the median number of ensemble sizes at their saturation were determined across all datasets, for each combination. The results are visualized as heatmaps in Fig. 10.

For predictive performance, RF and SVM ensembles reach the saturation earlier than the other techniques, independently of the featurization. Since RFs are already ensembles and SVMs are considered stable (i.e., showing little variance between individual SVM models), ensembles thereof will not provide much improvement, hence the saturation is reached after only a few members. For the SNN, it highly depends on the featurization. With MACCS and MFC, SNN ensembles require less members than when combined with RDKit descriptors or CDDD. Ensembles of XGB and DNN models show less variability in the size required to reach the saturation. In case of UQ performance, dependence on featurization or modeling techniques was less apparent. In contrast to predictive performances, there was no anti-correlation between ensemble sizes at saturation and full ensemble performances, but a slight correlation (see again Additional file 1: Fig. S6).

In some cases, ensembles turned out to be unsuccessful, i.e., they did not outperform single member models. Examples comprise cumulative member curves that remained constant, were too noisy to clearly detect an improvement even after the permutation approach, or even decreased. Ensembles ”fail entirely when bias is a dominant source of error” [15]. In such cases, quantifying the uncertainty using ensembles becomes meaningless. The inclusion of more members will then lead to no improvement or even to deterioration of the UQ performance compared to smaller ensembles. The lack of improvement is also reflected in those box-whisker plots in Fig. 9 where the saturation was detected already at the beginning of the fitted curve. One example for no improvement in UQ performance is using SNN ensembles and MFC to model the dataset F7. This should encourage practitioners to always validate the success of ensembles by visualizing cumulative member curves.

Despite the effort to cover most common practices in cheminformatic ML, the conclusion drawn might not hold for other sampling strategies, modeling techniques, and/or featurization methods that were not addressed here.

### Benchmarking DNN ensembles against single DNNs

**Fig. 11:**
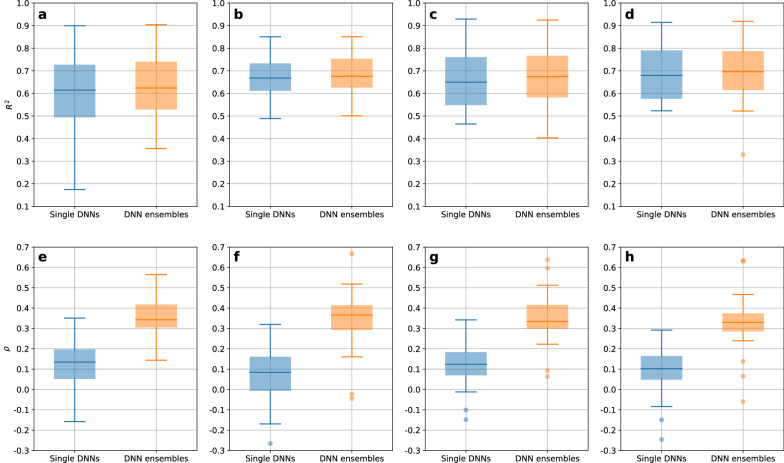
Comparisons between single DNNs and DNN ensembles of 200 members obtained by 2-fold CV. Single DNN performances are colored in blue, performances by DNN ensembles in orange. The upper row shows the comparisons between predictive performances ($R^2$) for MACCS (**a**), MFC (**b**), RDKit descriptors (**c**), and CDDD (**d**). The lower row shows the comparisons between UQ performances ($\rho$) for MACCS (**e**), MFC (**f**), RDKit descriptors (**g**), and CDDD (**h**)

To evaluate how subsampling ensembles perform in comparison to the aforementioned built-in mechanisms for UQ, DNN ensembles of 200 2-fold CV members were benchmarked against single model DNNs. Single model DNN uncertainty was quantified using MC dropout. To perform MC dropout, each test object was predicted with 100 different dropout masks. From the resulting 100 predictions per object, the final prediction was obtained by averaging, the uncertainty was quantified by the standard deviation. To obtain predictions from single DNNs, a single 10-fold CV was run for each combination of datasets and featurizations. As can be seen in Fig. 11, the predictive performance of single DNN models is comparable to those of DNN ensembles. In direct comparison for each of the 128 cases (32 datasets * four featurizations), 23 achieved a higher $R^2$ than DNN ensembles in the single model case. The differences were mostly marginal, ranging from 0.001 up to 0.242, with a median of 0.009. For the other 105 cases in which DNN ensembles succeeded, the differences in $R^2$ ranged from below 0.001 to 0.181, with a median of 0.021. UQ performance was, on average, higher when using the ensemble uncertainty of 200 members. When comparing UQ for each dataset and featurization pairwise for both approaches, standard deviations of ensembles based on 200 2-fold CV members constantly outperformed MC dropout, with the exception of two cases (namely for IL4, using CDDD, and F7, using MACCS). The differences were again marginal ($\Delta \rho$ of 0.041 and 0.026, respectively). All single DNN predictive performances and UQ performances are visualized in Additional file 1: Fig. S7.

The findings that ensemble uncertainties are more effective than uncertainties obtained by MC dropout are consistent with the results that Scalia et al. presented for message passing neural networks [50]. A possible reason they consider is that DNN ensembles, in contrast to “weight-sharing” MC dropout ensembles, provide a better coverage of the function space which induces more diversity and thus leads to the observed result [51].

### Benchmarking RF ensembles against single RFs

**Fig. 12:**
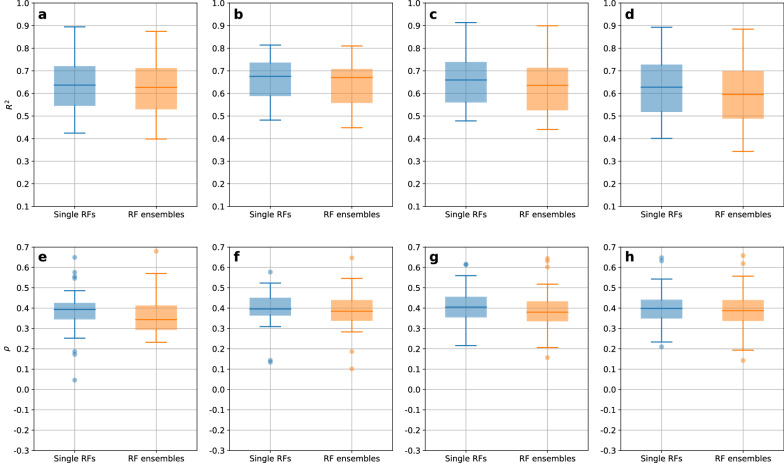
Comparisons between single RF models and RF ensembles of 200 members obtained by 2-fold CV. Single RF model performances are colored in blue, performances by RF ensembles in orange. The upper row shows the comparisons between predictive performances ($R^2$) for MACCS (**a**), MFC (**b**), RDKit descriptors (**c**), and CDDD (**d**). The lower row shows the comparisons between UQ performances ($\rho$) for MACCS (**e**), MFC (**f**), RDKit descriptors (**g**), and CDDD (**h**)

RF ensembles of 200 2-fold CV members were also benchmarked against single RF models (which are already ensembles) in terms of predictive performance and UQ performance. Predictions and uncertainties of a single RF correspond to the average and standard deviation, respectively, of the predictions of all decision trees which are members of the respective RF. To obtain predictions from single RFs, a single 10-fold CV was run for each combination of datasets and featurizations. Figure 12 outlines the performance comparison between single RF models and 200 2-fold CV RF ensembles. In contrast to the benchmark of ensembles against single models for DNNs, the success of one method over the other is less apparent for RFs. For predictive performance, ensembles succeeded closely in 12 out of 128 cases, with differences in $R^2$ between 0.002 and 0.074 and a median of 0.024. For UQ performance, single RFs succeeded in 75 out of 128 cases, exhibiting differences in $\rho$ from below 0.001 up to 0.149, with a median of 0.036. All single RF predictive performances and UQ performances are visualized in Additional file 1: Fig. S7.

It was expected that computing ensembles of an already ensemble-based method does neither harm nor improve its performance noticeably. The plots for most cumulative predictive performances confirm these observations, as the increase in $R^2$ was, in many cases, barely noticeable for RF ensembles (data not shown here, but visualized for all datasets and combinations in [44]). The marginal differences between the results of single models and ensembles are likely by chance.

## Conclusions

The following four main statements and recommendations are concluded from this study:

1. Practitioners who use ensembles for UQ should first check whether a single model benefits from more members, instead of trusting the consensus predictions and/or UQ without any further analysis. For that, visualizing the change in performance when accumulating more and more members proves beneficial.
2. When using ensembles to increase predictive performance, a smaller ensemble size suffices, while substantially more ensemble members are required for improving UQ performance.
3. The observation that UQ by subsampling ensembles prevails over UQ by MC dropout was already made for message-passing neural networks and is now also confirmed for DNNs in combination with various featurizations. For RFs, uncertainties obtained by ensembles performed comparably to the built-in uncertainties.
4. For techniques that do not provide UQ estimates by design, the applicability of UQ by ensembles could be demonstrated. While the method worked very well with XGB for predictive performance and best for UQ performance, regression SVMs and SNNs were usually the least successful techniques in this benchmark.

Because ensembles can be compiled for every modeling technique, they are expected to remain the default for UQ in regression. As an empirical study, the conclusions drawn are valid only for the settings studied.

## Methods

### Datasets

From the 32 datasets used in this study, 29 were taken from the supplementary information of a study by Cortes-Ciriano [41], of which 26 required additional filtering steps (considering only those that are of the ”Small molecule” type, with an assay score confidence of $\ge$ 8, and ”nM” as measured activity unit). The other three datasets, Tetrahymena [47], FreeSolv [48], and ESOL [52], are not related to activity data, but to inhibitory growth concentration, hydration free energies, and aqueous solubility, respectively. Some of the datasets are included in the DeepChem framework [53]. For the sake of consistency, all datasets were first exported (when coming from DeepChem) or converted (when extracted from a publication’s supplementary material) to table files containing SMILES before any filtering or featurization took place. After extracting the SMILES and their target values, each dataset was run through the recently published chemical structure curation pipeline [54] that was developed to standardize molecules before entering ChEMBL [55]. Steps of the pipeline include typical chemical data curation operations, e.g., removal of counterions and neutralization of ionized compounds. In the rare case where several isomers occurred in the same SMILES, stereoinformation was removed and only one structure was kept. In any case, only one connected SMILES was considered. Multiple outputs for a single compound were averaged. From the identifiers (mostly the compound ChEMBL ID) of the averaged entries, the first one was kept. Additional file 1: Table S1 lists all datasets, including their sizes before and after filtering.

### Molecular featurizations

#### Classical descriptors

The first three featurizations correspond to well-known structural and physicochemical descriptors, namely MACCS counts, circular fingerprint counts [56], and RDKit descriptors. All were computed using RDKit [39]. For MACCS counts, multiple substructure occurrences were summed up, due to performance improvement over the original MACCS binary vectors [38]. For convenience, they will be referred to as MACCS, although implying MACCS counts. When generating circular fingerprint counts, the GetHashedMorganFingerprint-function was called with an atom neighbor radius of three, while all other parameters were kept in their default state, resulting in integer vectors of length 2048. The occurrences per bin were added to obtain counts instead of binary vectors and are referred to as Morgan fingerprint count (MFC). RDKit descriptors were computed with all parameters on default, yielding a diverse collection of 208 physicochemical but also fragment-based descriptor values.

#### Learned representations

The pretrained CDDD encoder model from the associated GitHub repository was used [57], generating 512 continuous values between −1 and 1. Instead of running each input SMILES through the encoder once, every SMILES was randomized 100 times using 100 random permutations of the atom indices and the RenumberAtoms-function of RDKit to generate a varying non-canonical SMILES. Subsequently, the column average of the resulting 100 values was taken for each feature of each compound. This procedure was recommended by the creators of CDDD as it improves the performance when used for ML by enhancing generalization.

#### Feature scaling

For the training partition of each split, MACCS and MFC, being entirely count-based integer features, were normalized to unit norm (such that all values $\in [0, 1]$) prior to fitting. RDKit descriptors and CDDD were standardized such that all feature columns have a mean of 0 and a standard deviation of 1.

### Modeling algorithms

No hyperparameter tuning was involved to compare the models at their baseline performance.

#### Techniques not related to neural networks

Implementations of RFs and SVMs were taken from scikit-learn [58], with the default parameters. As RFs are already ensembles by construction, the ensembles of 200 members which were used in the conducted experiments correspond to ensembles of ensembles in case of RFs. For the implementation of XGB, the XGBoost Python Package was used [59].

#### Neural networks

The SNN architecture has 128 neurons in its hidden layer, while the DNN architecture has layers of 256, 128, and 16 neurons, in the direction of forward propagation. Each hidden layer uses ReLU activation. The output neuron uses a linear activation for regression. Modeling tasks related to neural networks were performed using TensorFlow [60]. To make the results comparable, dropout was used for all hidden layers in the DNN, with a dropout rate of 20%. All neural networks were trained for 1000 epochs.

### Cross-validation for generating subsampling ensembles

The size of ensembles generated by CV was set to 200 in order to lower the variance of the ensemble derived estimates ($R^2$, $\rho$). The number of repetitions was determined by preliminary experiments (data not shown here), where several settings were tested on a few selected datasets. A custom implementation was built for convenient evaluation of sampling-based ensemble uncertainties [61]. An overview of each Python package, together with its version number, can be found in Additional file 1: Table S2.

A balanced splitting scheme, i.e., each test object is in the test set exactly once, allows for estimation of the model quality with lower variability, which is why *k*-fold CV  [62] was applied to generate subsamples for ensemble construction. To ensure diversity of the resulting ensemble members on the one hand but also reasonable model quality on the other, *k* was set to 2. This choice has also turned out to be successful in the preliminary experiments, requiring less CPU time than larger *k*s and showing a comparable model performance at the same time. Therefore, 400 (= 200 repetitions * two folds) models were fitted and evaluated per dataset, featurization, and modeling technique. The complete prediction of all compounds in a dataset requires two folds (i.e., two models).

Performance measures based on random sampling from the entire dataset do not account for specific shifts in the chemical space, i.e., shifts that may occur in project work. The impact of such shifts on predictive and UQ performance can only be estimated when time stamps for each molecule in the dataset are available, e.g., by splitting the dataset in training and test set based on the time stamps (so-called time splits). Usually, predicting the outputs of actual future compounds turns out considerably harder than predicting compounds of a random subsample. Since no reliable time stamps were available for the benchmark datasets used, the more realistic simulation of future predictions by time splits was unfortuantely not possible here. It should be recalled that changing the sampling scheme for subsampling likely changes the results, in particular, if the sampling schemes differ substantially.

### Benchmarking uncertainty measures

For subsampling ensembles, the standard deviation of ensemble predictions was computed across the predictions of all members to quantify the ensemble uncertainty. The two modeling techniques that come with specific uncertainty measures were RFs and DNNs, namely with the standard deviation of the individual decision trees of the respective RF, and the standard deviation of MC dropout predictions. These two algorithms were also evaluated in addition to the ensembles generated by subsampling. Hereby, a single 10-fold CV was performed to produce one prediction per compound, with again all other parameters on default settings. The default technique-specific UQ measure was used for this single prediction. In the case of DNNs, MC dropout was implemented with dropout in every hidden layer and a dropout rate of 20%. The output of each compound was predicted 100 times with different weights randomly dropped out of the fitted network.

### Quality assessment

The predictive performance was assessed by $R^2_{test}$ (the cross-validated coefficient of determination, abbreviated as $R^2$ here) as it is a relative metric that allows for comparison between different models [63]. Since hyperparameter tuning was omitted, CV corresponds to actual estimation of test set predictivity. $R^2$ can also become negative, namely in cases where the sum of squares of prediction errors becomes larger than the total sum of squares.

Predictions of higher uncertainty are expected to be accompanied by larger prediction errors. UQ performance was therefore determined by the ability of the uncertainty measure to rank the predictions according to the magnitude in error. A ranking metric that was successfully applied to evaluate UQ is Spearman’s correlation coefficient $\rho$ [14]. Effectively, $\rho$ measures the order similarity between two lists of ranks by computing Pearson’s correlation coefficient on the ranks instead of on the raw values. A perfect rank correlation is indicated by 1, where a perfect anti-correlation will yield a $\rho$ of $-1$. For uncorrelated data, $\rho$ will be close to 0. Even though the coefficient meaningfully captures the ranking ability, values of $\rho$ appear rather low. Due to the assumed normal distribution of prediction errors, a perfect ranking ability of $\rho = 1$ is unrealistic [14].

### Growing ensembles for cumulative member curves

The changes in $R^2$ and $\rho$ were inspected member-wise. Hereby, more and more members were aggregated, starting with only the first one, then the first and the second one, and so on, resulting in cumulative values for $R^2$. For the evaluation of $\rho$, the number of members starts at two, as it is the minimum required number to calculate standard deviations. The arithmetic mean served as the single-valued prediction at each step, and the standard deviation as UQ measure. However, the level of improvement might be biased by the order in which the members were generated, i.e., the random seed. To remove this potential bias and to give an estimate of the average performance at each ensemble size, the 200 members were shuffled 200 times and the cumulative member curves for each setting were computed from each of the shuffled orders, followed by taking the average performance at each ensemble size. Additionally to giving an estimation of the median performance that can be achieved for a given ensemble size and setting, this procedure also smooths the curves. Next, a Michaelis–Menten function [64, 65] was fitted through each median curve. Although the data were not generated by enzymatic kinetics, the Michaelis–Menten function fits the median curves well and allows for an automated detection of the saturation. The first member-wise step in the fitted curve from one size to the next that yielded a performance gain below 0.0001 was considered the point of saturation. The threshold of 0.0001 expresses the point where the improvement becomes irrelevantly small, namely making changes in the fourth decimal place.

## Supplementary Information

**Additional file 1: Figure S1.** Overview of the predictive performances (*R*^*2*^) of the 200-member ensembles, for all datasets and each combination of featurization and modeling technique. Brighter colors correspond to larger values. **Figure S2.1.** Differences in predictive performances between subsampling by 2-fold CV and by bootstrapping for the eight selected datasets. Each row corresponds to the comparisons between results of one dataset, each plot in a row shows the results for a specific modeling technique. Within each plot, a pair of bars represents the results for a specific featurization, also indicated by the color. The left bar of each pair shows *R*^*2*^ when generating 200 members by 2-fold, the right bar when using bootstrapping. **Figure S2.2.** Differences in UQ performances between subsampling by 2-fold CV and by bootstrapping for the eight selected datasets. Each row corresponds to the comparisons between results of one dataset, each plot in a row shows the results for a specific modeling technique. Within each plot, a pair of bars represents the results for a specific featurization, also indicated by the color. The left bar of each pair shows *ρ* when generating 200 members by 2-fold, the right bar when using bootstrapping. **Figure S3.1.** Raw predictions. **Figure S3.2.** Predictions after removing those that are outside the acceptable interval. **Figure S3.3.** Predictions when removing ’Kappa3’ from the dataset featurized as RDKit descriptors before machine learning. **Figure S4.** Overview of the UQ performances (*ρ*) of the 200-member ensembles, for all datasets and each combination of featurization and modeling technique. Brighter colors correspond to larger values. **Figure S5.1.** Raw cumulative member curves for predictive performance (**a**) and UQ performance (**b**), for all descriptors, modeling P03372, using SNN ensembles. **Figure S5.2.** Median cumulative member curves for predictive performance (**a**) and UQ performance (**b**), for all descriptors, obtained from 200 permutations of the 200 members per setting. **Figure S5.3.** Michaelis-Menten functions, fitted to the median cumulative member curves for predictive performance (**a**) and UQ performance (**b**), for all descriptors. The median cumulative member curves are shown as thin black lines. The estimated saturation point for each fitted curve (i.e., the first ensemble size where the corresponding gain in performance falls below 0.0001) is depicted as vertical bar. **Figure S6.** Median ensembles at saturation against full ensemble performance, for predictive performance (**a**) and UQ performance (**b**), for all datasets (32 points in each plot). **Figure S7.** Overview of the predictive performances (*R*^*2*^) and UQ performances (*ρ*) of the 10-fold CV single DNN models, for all datasets and each featurization. Brighter colors correspond to larger values. **Figure S8.** Overview of the predictive performances (*R*^*2*^) and UQ performances (*ρ*) of the 10-fold CV single RF models, for all datasets and each featurization. Brighter colors correspond to larger values. **Table S1.** All data sets used for evaluation. The original number of compounds refers to the number of measurement points in the raw files, the number of compounds after pipeline to the preprocessed files of which descriptor values were computed from. **Table S2.** Python packages with versions used for this study.

## Data Availability

The framework to evaluate ensemble uncertainties is available in the corresponding repository [61]. Datasets, results, scripts, and the Jupyter notebooks to obtain the visualizations and statistics from the results presented within this study are available in [66].

## Abbreviations

CDDD: Continuous and data-driven descriptor
CV: Cross-validation
DL: Deep learning
DNN: Deep neural network
MC: Monte Carlo
MFC: Morgan fingerprint count
ML: Machine learning
$R^2$: Coefficient of determination
RF: Random forest
$\rho$: Spearman’s rank correlation coefficient
SNN: Shallow neural network
SVM: Support vector machine
UQ: Uncertainty quantification
XGB: XGBoost

## References

[1] Vamathevan J, Clark D, Czodrowski P, Dunham I, Ferran E, Lee G, Li B, Madabhushi A, Shah P, Spitzer M (2019). Applications of machine learning in drug discovery and development. Nat Rev Drug Discov.

[2] Chen H, Engkvist O, Wang Y, Olivecrona M, Blaschke T (2018). The rise of deep learning in drug discovery. Drug Discov Today.

[3] Tropsha A, Gramatica P, Gombar VK (2003). The importance of being earnest: validation is the absolute essential for successful application and interpretation of qspr models. SAR Comb Sci.

[4] Netzeva TI, Worth AP, Aldenberg T, Benigni R, Cronin MT, Gramatica P, Jaworska JS, Kahn S, Klopman G, Marchant CA (2005). Current status of methods for defining the applicability domain of (quantitative) structure-activity relationships: the report and recommendations of ECVAM Workshop 52. Altern Lab Anim.

[5] Mervin LH, Johansson S, Semenova E, Giblin KA, Engkvist O (2021). Uncertainty quantification in drug design. Drug Discov Today.

[6] Kiureghian AD, Ditlevsen O (2009). Aleatory or epistemic? Does it matter?. Struct Saf.

[7] Tagasovska N, Lopez-Paz D, Single-model uncertainties for deep learning. 10.48550/arXiv.1811.00908

[8] Mathea M, Klingspohn W, Baumann K (2016). Chemoinformatic classification methods and their applicability domain. Mol Inf.

[9] Platt J (1999) Probabilistic outputs for support vector machines and comparisons to regularized likelihood methods. In: Smola AJ, Bartlett P, Schölkopf B, Schuurmans D (eds) Advances in large margin classifiers. MIT Press, Cambridge, MA, pp 61–72

[10] Cortes C, Vapnik V (1995). Support-vector networks. Support-vector networks. Machine learningMach Learn.

[11] Drucker H, Burges CJC, Kaufman L, Smola A, Vapnik V (1996) Support vector regression machines. In: Mozer M, Jordan M, Petsche T (eds) Advances in neural information processing systems, MIT Press, Cambridge, MA, vol 9, pp 155–161. https://proceedings.neurips.cc/paper/1996/file/d38901788c533e8286cb6400b40b386d-Paper.pdf

[12] Dietterich T (2000) Ensemble methods in machine learning. In: Lecture Notes in Computer Science 1857, International Workshop on Multiple Classifier Systems, Cagliari, Italy, 21–23 June 2000, pp 1–15, 10.1007/3-540-45014-9_1

[13] Lakshminarayanan B, Pritzel A, Blundell C (2017) Simple and scalable predictive uncertainty estimation using deep ensembles. In: Proceedings of the 31st International Conference on Neural Information Processing Systems, Curran Associates Inc., Red Hook, NY, USA, NIPS’17, pp 6405–6416

[14] Hirschfeld L, Swanson K, Yang K, Barzilay R, Coley CW (2020). Uncertainty quantification using neural networks for molecular property prediction. J Chem Inf Model.

[15] Palmer G, Du S, Politowicz A, Emory JP, Yang X, Gautam A, Gupta G, Li Z, Jacobs R, Morgan D (2022). Calibration after bootstrap for accurate uncertainty quantification in regression models. NPJ Comput Mater.

[16] Hüllermeier E, Waegeman W (2021). Aleatoric and epistemic uncertainty in machine learning: an introduction to concepts and methods. Mach Learn.

[17] Breiman L (2001). Random forests. Mach Learn.

[18] Dutschmann TM, Baumann K (2021). Evaluating high-variance leaves as uncertainty measure for random forest regression. Molecules.

[19] Srivastava N, Hinton G, Krizhevsky A, Sutskever I, Salakhutdinov R (2014) Dropout: a simple way to prevent neural networks from overfitting. J Mach Learn Res 15:1929–1958. https://jmlr.org/papers/v15/srivastava14a.html

[20] Gal Y, Ghahramani Z (2016) Dropout as a Bayesian approximation: representing model uncertainty in deep learning. In: International conference on machine learning, PMLR, New York, New York, USA, Proceedings of Machine Learning Research, vol 48, pp 1050–1059. http://proceedings.mlr.press/v48/gal16.pdf

[21] Hara K, Saitoh D, Shouno H, Analysis of dropout learning regarded as ensemble learning. 10.48550/arXiv.1706.06859

[22] Cortes-Ciriano I, Bender A (2019). Reliable prediction errors for deep neural networks using test-time dropout. J Chem Inf Model.

[23] Kimber TB, Gagnebin M, Volkamer A (2021). Maxsmi: maximizing molecular property prediction performance with confidence estimation using smiles augmentation and deep learning. Artif Intell Life Sci.

[24] Wang D, Yu J, Chen L, Li X, Jiang H, Chen K, Zheng M, Luo X (2021). A hybrid framework for improving uncertainty quantification in deep learning-based qsar regression modeling. J Cheminform.

[25] Abdar M, Pourpanah F, Hussain S, Rezazadegan D, Liu L, Ghavamzadeh M, Fieguth P, Cao X, Khosravi A, Acharya UR, Makarenkov V, Nahavandi S (2021). A review of uncertainty quantification in deep learning: techniques, applications and challenges. Inform Fusion.

[26] Soleimany AP, Amini A, Goldman S, Rus D, Bhatia SN, Coley CW (2021). Evidential deep learning for guided molecular property prediction and discovery. ACS Cent Sci.

[27] Pearce T, Leibfried F, Brintrup A (2020) Uncertainty in neural networks: approximately bayesian ensembling. In: International conference on artificial intelligence and statistics, PMLR, pp 234–244. http://proceedings.mlr.press/v108/pearce20a/pearce20a.pdf

[28] Grisoni F, Consonni V, Todeschini R (2018) Impact of molecular descriptors on computational models. In: Computational chemogenomics, Springer, Humana Press, New York, NY, pp 171–209. 10.1007/978-1-4939-8639-2_5 10.1007/978-1-4939-8639-2_5 30334206

[29] Raghunathan S, Priyakumar UD (2021) Molecular representations for machine learning applications in chemistry. Int J Quantum Chem e26870. 10.1002/qua.26870

[30] Consonni V, Todeschini R (2010) Molecular Descriptors. In: Recent advances in QSAR studies, Springer, Springer, Dordrecht, pp 29–102. 10.1007/978-1-4020-9783-6_3

[31] Gómez-Bombarelli R, Wei JN, Duvenaud D, Hernández-Lobato JM, Sánchez-Lengeling B, Sheberla D, Aguilera-Iparraguirre J, Hirzel TD, Adams RP, Aspuru-Guzik A (2018). Automatic chemical design using a data-driven continuous representation of molecules. ACS Cent Sci.

[32] Hwang D, Yang S, Kwon Y, Lee KH, Lee G, Jo H, Yoon S, Ryu S (2020). Comprehensive study on molecular supervised learning with graph neural networks. J Chem Inf Model.

[33] Yang K, Swanson K, Jin W, Coley C, Eiden P, Gao H, Guzman-Perez A, Hopper T, Kelley B, Mathea M (2019). Analyzing learned molecular representations for property prediction. J Chem Inf Model.

[34] Winter R, Montanari F, Noé F, Clevert DA (2019). Learning continuous and data-driven molecular descriptors by translating equivalent chemical representations. Chem Sci.

[35] Svensson F, Aniceto N, Norinder U, Cortes-Ciriano I, Spjuth O, Carlsson L, Bender A (2018). Conformal regression for quantitative structure-activity relationship modeling-quantifying prediction uncertainty. J Chem Inf Model.

[36] Zhang Y (2019). Bayesian semi-supervised learning for uncertainty-calibrated prediction of molecular properties and active learning. Chem Sci.

[37] Busk J, Jørgensen PB, Bhowmik A, Schmidt MN, Winther O, Vegge T (2021). Calibrated uncertainty for molecular property prediction using ensembles of message passing neural networks. Mach Sci Technol.

[38] Durant JL, Leland BA, Henry DR, Nourse JG (2002). Reoptimization of MDL keys for use in drug discovery. J Chem Inf Comp Sci.

[39] Landrum G, RDKit: open-source cheminformatics software. https://www.rdkit.org. Accessed 16 Mar 2022

[40] Chen T, Guestrin C (2016) XGBoost: A scalable tree boosting system. In: Proceedings of the 22nd acm sigkdd international conference on knowledge discovery and data mining, pp 785–794. 10.1145/2939672.2939785

[41] Cortes-Ciriano I (2016). Benchmarking the predictive power of ligand efficiency indices in qsar. J Chem Inf Model.

[42] Koutsoukas A, Monaghan KJ, Li X, Huan J (2017). Deep-learning: investigating deep neural networks hyper-parameters and comparison of performance to shallow methods for modeling bioactivity data. J Cheminf.

[43] Murphy KP (2012). Machine learning: a probabilistic perspective.

[44] Dutschmann TM, Kinzel L, Cumulative curves for growing ensembles. https://git.rz.tu-bs.de/impc/baumannlab/supporting-repository-for-ensemble-publication/-/tree/main/data/generated_by_notebooks/plots/permutated_cumulative_members_curve_plots. Accessed 25 Feb 2023

[45] Balfer J, Bajorath J (2015). Systematic artifacts in support vector regression-based compound potency prediction revealed by statistical and activity landscape analysis. PLOS ONE.

[46] Rodriguez-Perez R, Vogt M, Bajorath J (2017). Support vector machine classification and regression prioritize different structural features for binary compound activity and potency value prediction. ACS Omega.

[47] Cheng F, Shen J, Yu Y, Li W, Liu G, Lee PW, Tang Y (2011). In silico prediction of Tetrahymena pyriformis toxicity for diverse industrial chemicals with substructure pattern recognition and machine learning methods. Chemosphere.

[48] Mobley DL, Guthrie JP (2014). FreeSolv: a database of experimental and calculated hydration free energies, with input files. J Comput-Aided Mol Des.

[49] Maggiora GM (2006). On outliers and activity cliffs—why QSAR often disappoints. J Chem Inf Model.

[50] Scalia G, Grambow CA, Pernici B, Li YP, Green WH (2020). Evaluating scalable uncertainty estimation methods for deep learning-based molecular property prediction. J Chem Inf Model.

[51] Fort S, Hu H, Lakshminarayanan B (2019) Deep ensembles: A loss landscape perspective. arXiv preprint arXiv:1912.02757. 10.48550/arXiv.1912.02757

[52] Delaney JS (2004). ESOL: estimating aqueous solubility directly from molecular structure. J Chem Inf Comp Sci.

[53] Ramsundar B, Eastman P, Walters P, Pande V (2019) Deep learning for the life sciences: applying deep learning to genomics, microscopy, drug discovery, and more. O’Reilly Media, Sebastopol, CA

[54] Bento AP, Hersey A, Félix E, Landrum G, Gaulton A, Atkinson F, Bellis LJ, De Veij M, Leach AR (2020). An open source chemical structure curation pipeline using RDKit. J Cheminf.

[55] Mendez D, Gaulton A, Bento AP, Chambers J, De Veij M, Félix E, Magariños MP, Mosquera JF, Mutowo P, Nowotka M (2019). ChEMBL: towards direct deposition of bioassay data. Nucleic Acids Res.

[56] Morgan HL (1965). The generation of a unique machine description for chemical structures—a technique developed at chemical abstracts service. J Chem Doc.

[57] Winter RL (2022) Continuous and data-driven descriptors (cddd). https://github.com/jrwnter/cddd. Accessed 16 Mar

[58] Pedregosa F, Varoquaux G, Gramfort A, Michel V, Thirion B, Grisel O, Blondel M, Prettenhofer P, Weiss R, Dubourg V, Vanderplas J, Passos A, Cournapeau D, Brucher M, Perrot M, Duchesnay E (2011) Scikit-learn: machine learning in Python. J Mach Learn Res 12:2825–2830, https://www.jmlr.org/papers/volume12/pedregosa11a/pedregosa11a.pdf

[59] XGBoost Developers (2022) Xgboost python package. https://xgboost.readthedocs.io/en/stable/python/. Accessed 17 Mar

[60] Abadi M, Agarwal A, Barham P, Brevdo E, Chen Z, Citro C, Corrado GS, Davis A, Dean J, Devin M, Ghemawat S, Goodfellow I, Harp A, Irving G, Isard M, Jia Y, Jozefowicz R, Kaiser L, Kudlur M, Levenberg J, Mané D, Monga R, Moore S, Murray D, Olah C, Schuster M, Shlens J, Steiner B, Sutskever I, Talwar K, Tucker P, Vanhoucke V, Vasudevan V, Viégas F, Vinyals O, Warden P, Wattenberg M, Wicke M, Yu Y, Zheng X, TensorFlow: Large-scale machine learning on heterogeneous systems. https://www.tensorflow.org/, software available from tensorflow.org

[61] Dutschmann TM, Kinzel L (2022) ensemble_uncertainties: Framework to evaluate predictive uncertainties by generating k-fold cross-validation ensembles. https://git.rz.tu-bs.de/impc/baumannlab/ensemble_uncertainties. Accessed 2 Aug

[62] Baumann D, Baumann K (2014). Reliable estimation of prediction errors for QSAR models under model uncertainty using double cross-validation. J Cheminf.

[63] Kvålseth TO (1985). Cautionary note about $$\rm r ^{2}$$. Am Stat.

[64] Michaelis L, Menten M (1913). Die Kinetik der Invertinwirkung. Biochem Z.

[65] Johnson KA, Goody RS (2011). The original Michaelis Constant: translation of the 1913 Michaelis-Menten Paper. Biochemistry.

[66] Dutschmann TM, Kinzel L (2023) Supporting Repository for "Large-scale evaluation of k-fold cross-validation ensembles for uncertainty estimation". https://git.rz.tu-bs.de/impc/baumannlab/supporting-repository-for-ensemble-publication/. Accessed 25 Feb. 10.1186/s13321-023-00709-9 PMC10142532 37118768

