# Supplementary material for: Large-scale evaluation of k-fold cross-validation ensembles for uncertainty estimation
Source: J Cheminform. 2023 Apr 28;15:49. doi: 10.1186/s13321-023-00709-9 (PMC10142532; doi:10.1186/s13321-023-00709-9)
Supplement: Supplementary file 1 — Additional file 1: Figure S1. Overview of the predictive performances (R2) of the 200-member ensembles, for all datasets and each combination of featurization and modeling technique. Brighter colors correspond to larger values. Figure S2.1. Differences in predictive performances between subsampling by 2-fold CV and by bootstrapping for the eight selected datasets. Each row corresponds to the comparisons between results of one dataset, each plot in a row shows the results for a specific modeling technique. Within each plot, a pair of bars represents the results for a specific featurization, also indicated by the color. The left bar of each pair shows R2 when generating 200 members by 2-fold, the right bar when using bootstrapping. Figure S2.2. Differences in UQ performances between subsampling by 2-fold CV and by bootstrapping for the eight selected datasets. Each row corresponds to the comparisons between results of one dataset, each plot in a row shows the results for a specific modeling technique. Within each plot, a pair of bars represents the results for a specific featurization, also indicated by the color. The left bar of each pair shows ρ when generating 200 members by 2-fold, the right bar when using bootstrapping. Figure S3.1. Raw predictions. Figure S3.2. Predictions after removing those that are outside the acceptable interval. Figure S3.3. Predictions when removing ’Kappa3’ from the dataset featurized as RDKit descriptors before machine learning. Figure S4. Overview of the UQ performances (ρ) of the 200-member ensembles, for all datasets and each combination of featurization and modeling technique. Brighter colors correspond to larger values. Figure S5.1. Raw cumulative member curves for predictive performance (a) and UQ performance (b), for all descriptors, modeling P03372, using SNN ensembles. Figure S5.2. Median cumulative member curves for predictive performance (a) and UQ performance (b), for all descriptors, obtained from 200 permutations of the 20 [file 13321_2023_709_MOESM1_ESM.pdf]

Additional information for: Large-scale evaluation of k-fold cross-validation ensembles for uncertainty estimation

Thomas-Martin Dutschmann†, Lennart Kinzel†, Antonius ter Laak‡, and Knut Baumann†

† *Institute of Medicinal and Pharmaceutical Chemistry, University of Technology Braunschweig, Beethovenstraße 55, 38106 Braunschweig, Germany*
‡ *Bayer AG, Research & Development, Pharmaceuticals, Muellerstrasse 178, D-13353 Berlin, Germany*

E-mail: t.dutschmann@tu-braunschweig.de, l.kinzel@tu-braunschweig.de, antoniuster.laak@bayer.com, k.baumann@tu-braunschweig.de

Contents

1 Full ensemble predictive performances . . . **S3**

2 Comparing 2-fold CV to bootstrapping . . . **S4**

3 Dealing with extreme prediction outliers . . . **S7**

4 Full ensemble UQ performances . . . **S8**

5 Obtaining the point of saturation from cumulative member curves . . . **S9**

6 Ensemble sizes at saturation vs. full ensemble performances . . . **S11**

7 Single DNN performances . . . **S12**

8 Single RF performances . . . **S13**

9 Summary of all data sets . . . **S14**

10 Python packages with versions . . . **S15**

11 Additional references . . . **S15**

1 Full ensemble predictive performances

	RF				XGB				SVM				SNN				DNN			
	MACCS	MFC	RDKit	CDDD	MACCS	MFC	RDKit	CDDD	MACCS	MFC	RDKit	CDDD	MACCS	MFC	RDKit	CDDD	MACCS	MFC	RDKit	CDDD
Tetrahymena	0.73	0.71	0.78	0.74	0.79	0.77	0.83	0.78	0.71	0.72	0.78	0.80	0.79	0.78	0.86	0.86	0.78	0.74	0.85	0.85
FreeSolv	0.87	0.68	0.89	0.84	0.90	0.76	0.91	0.87	0.65	0.45	0.70	0.65	0.88	0.79	0.93	0.93	0.90	0.68	0.92	0.91
ESOL	0.78	0.71	0.90	0.88	0.82	0.76	0.91	0.90	0.66	0.61	0.85	0.88	0.81	0.76	0.89	0.93	0.79	0.74	0.91	0.92
MMP2	0.43	0.47	0.44	0.45	0.48	0.53	0.48	0.47	0.15	0.40	0.39	0.46	0.19	0.58	-0.26	0.36	0.07	0.50	0.40	0.33
IL4	0.50	0.50	0.49	0.45	0.52	0.59	0.56	0.50	0.24	0.45	0.51	0.54	0.31	0.61	0.09	0.24	0.48	0.61	0.57	0.57
F7	0.65	0.70	0.70	0.69	0.69	0.73	0.72	0.72	0.46	0.59	0.67	0.71	0.50	0.70	0.37	0.42	0.36	0.64	0.69	0.69
O60674	0.49	0.54	0.49	0.47	0.52	0.60	0.52	0.52	0.26	0.50	0.49	0.55	0.36	0.57	0.51	0.54	0.52	0.61	0.55	0.59
O14965	0.61	0.69	0.60	0.57	0.65	0.73	0.65	0.63	0.35	0.62	0.57	0.65	0.48	0.70	0.62	0.70	0.67	0.75	0.68	0.72
P03372	0.67	0.69	0.70	0.67	0.69	0.72	0.73	0.70	0.54	0.64	0.66	0.70	0.59	0.69	0.67	0.72	0.69	0.73	0.71	0.73
P04150	0.62	0.63	0.64	0.62	0.65	0.66	0.67	0.65	0.51	0.60	0.63	0.67	0.60	0.62	0.67	0.68	0.63	0.67	0.67	0.69
P06401	0.69	0.70	0.69	0.64	0.71	0.74	0.71	0.68	0.52	0.68	0.64	0.70	0.61	0.73	0.70	0.72	0.71	0.75	0.72	0.74
P11229	0.71	0.69	0.69	0.64	0.73	0.71	0.71	0.67	0.56	0.61	0.67	0.70	0.63	0.66	0.66	0.72	0.73	0.72	0.73	0.75
P12931	0.69	0.72	0.69	0.65	0.72	0.75	0.73	0.70	0.48	0.67	0.64	0.71	0.59	0.70	0.70	0.74	0.68	0.76	0.73	0.75
P16581	0.59	0.68	0.64	0.60	0.58	0.70	0.62	0.62	0.48	0.65	0.54	0.63	0.53	0.62	0.53	0.58	0.57	0.65	0.61	0.68
P17252	0.64	0.65	0.62	0.58	0.65	0.68	0.65	0.61	0.43	0.57	0.58	0.62	0.46	0.63	0.61	0.61	0.62	0.67	0.66	0.65
P18089	0.55	0.56	0.59	0.56	0.55	0.57	0.59	0.58	0.49	0.57	0.51	0.60	0.47	0.52	0.32	0.23	0.50	0.61	0.51	0.54
P19327	0.53	0.57	0.53	0.49	0.56	0.64	0.57	0.54	0.34	0.53	0.51	0.56	0.46	0.62	0.56	0.62	0.54	0.66	0.59	0.63
P21554	0.68	0.72	0.70	0.69	0.72	0.77	0.74	0.73	0.51	0.69	0.73	0.75	0.60	0.76	0.77	0.80	0.74	0.80	0.79	0.81
P24530	0.73	0.77	0.76	0.72	0.77	0.81	0.79	0.77	0.53	0.69	0.68	0.75	0.63	0.80	0.78	0.83	0.75	0.83	0.80	0.83
P25929	0.74	0.76	0.74	0.71	0.75	0.78	0.76	0.74	0.45	0.64	0.64	0.72	0.50	0.72	0.72	0.73	0.74	0.82	0.80	0.81
P28335	0.53	0.54	0.52	0.49	0.55	0.58	0.56	0.53	0.29	0.47	0.46	0.55	0.38	0.51	0.50	0.57	0.54	0.59	0.58	0.62
P28482	0.40	0.45	0.45	0.34	0.43	0.55	0.52	0.39	0.15	0.29	0.34	0.36	0.22	0.54	0.18	0.28	0.53	0.63	0.59	0.56
P35968	0.60	0.63	0.58	0.52	0.63	0.66	0.64	0.61	0.31	0.58	0.55	0.64	0.50	0.66	0.63	0.69	0.61	0.70	0.65	0.70
P41594	0.47	0.55	0.48	0.46	0.47	0.60	0.51	0.51	0.29	0.50	0.43	0.53	0.38	0.56	0.46	0.59	0.47	0.60	0.50	0.61
P42345	0.78	0.80	0.77	0.75	0.80	0.82	0.80	0.78	0.61	0.77	0.76	0.80	0.71	0.82	0.81	0.83	0.79	0.84	0.81	0.84
P47871	0.57	0.60	0.60	0.59	0.58	0.66	0.63	0.61	0.36	0.51	0.59	0.63	0.38	0.62	0.62	0.66	0.54	0.63	0.63	0.66
P49146	0.72	0.73	0.74	0.71	0.74	0.76	0.74	0.73	0.63	0.72	0.67	0.74	0.62	0.67	0.70	0.70	0.75	0.76	0.76	0.78
P61169	0.48	0.56	0.48	0.45	0.51	0.61	0.54	0.50	0.31	0.52	0.45	0.53	0.39	0.57	0.51	0.57	0.51	0.63	0.55	0.59
Q05397	0.41	0.48	0.47	0.41	0.45	0.50	0.51	0.46	0.25	0.40	0.41	0.44	0.25	0.45	0.33	0.42	0.43	0.53	0.49	0.52
Q16602	0.78	0.81	0.82	0.80	0.81	0.84	0.84	0.82	0.59	0.74	0.79	0.82	0.68	0.82	0.85	0.86	0.79	0.85	0.86	0.87
P24941	0.63	0.67	0.64	0.57	0.65	0.71	0.66	0.63	0.47	0.64	0.61	0.67	0.53	0.70	0.65	0.69	0.65	0.73	0.68	0.72
Q92731	0.57	0.56	0.59	0.55	0.60	0.62	0.62	0.59	0.38	0.49	0.55	0.61	0.47	0.60	0.60	0.66	0.57	0.63	0.62	0.67

Figure S1: Overview of the predictive performances (R^2) of the 200-member ensembles, for all datasets and each combination of featurization and modeling technique. Brighter colors correspond to larger values.

2 Comparing 2-fold CV to bootstrapping

For a selection of eight datasets, another 200 members were generated using the bootstrapping procedure instead of 2-fold CV to generate the random subsamples. Producing repetitive bootstrap samples of dataset size n and averaging over all predictions for each left out object is also known as leave-one-out bootstrap [S1, S2]. Leave-one-out bootstrap was found to yield error rates comparable to 2-fold CV (called "half-sample CV" in the original publication) [S3], which raised the question whether this comparability also holds for predictive performance and UQ performance. To make potential differences noticeable, the comparison between the two subsampling techniques was surveyed for datasets with performances of exceptional dependency on modeling technique or featurization. Overall, the results were comparable, both methods exhibited the same strengths and weaknesses within a dataset and a given combination of featurization and modeling technique. While bootstrap ensembles slightly outperformed 2-fold CV ensembles for predictive performance, they were surpassed for UQ performance. The performance comparisons are visualized in Fig. S2.1 and S2.2, for predictive performance and UQ performance, respectively. Since the training sample in the bootstrap approach covers a larger fraction of objects, the individual ensemble members are expectedly slightly less diverse. This might improve the predictive quality due to the better information coverage, but leads to less variance to estimate uncertainties at the same time.

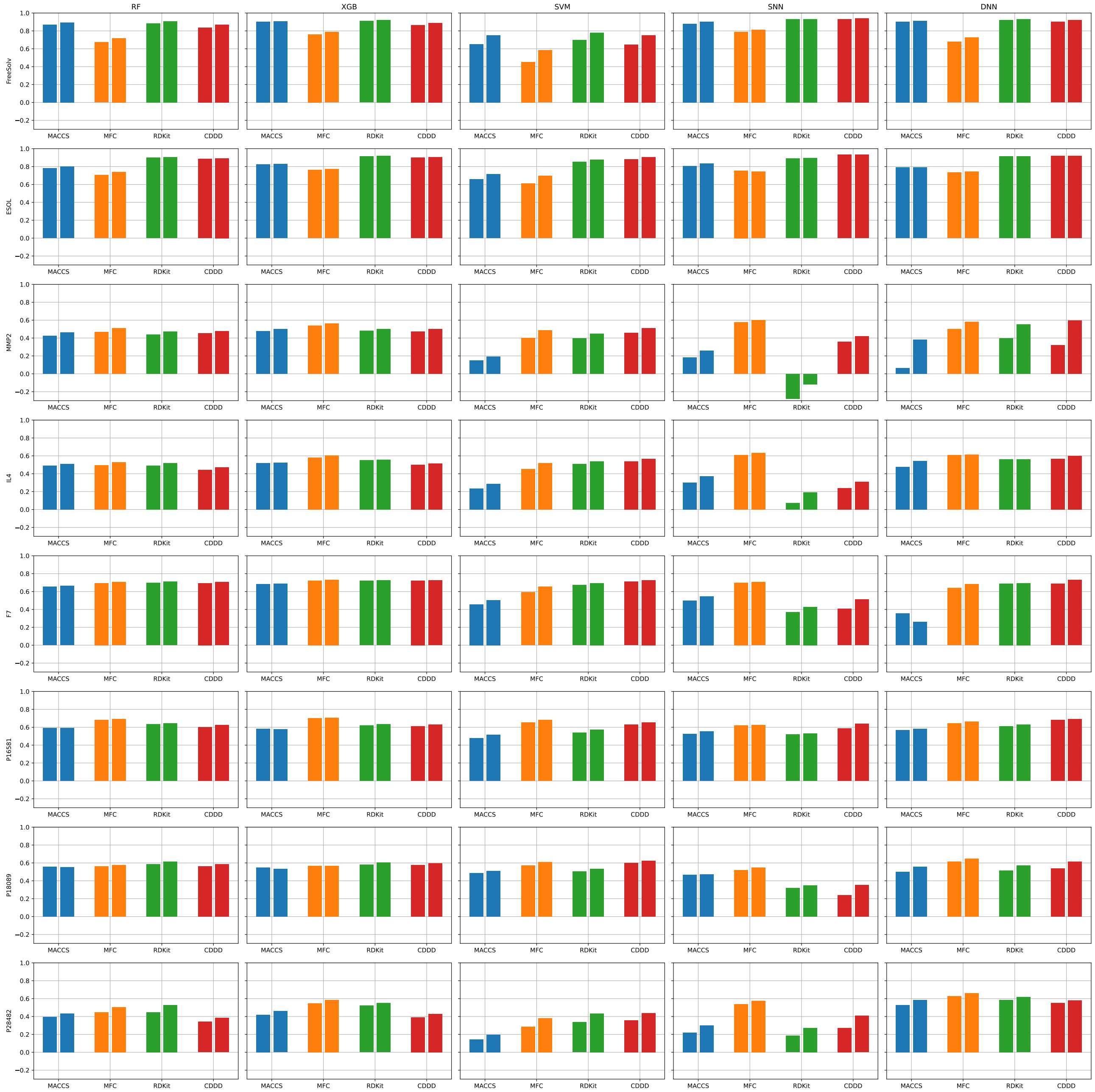

Figure S2.1: Differences in predictive performances between subsampling by 2-fold CV and by bootstrapping for the eight selected datasets. Each row corresponds to the comparisons between results of one dataset, each plot in a row shows the results for a specific modeling technique. Within each plot, a pair of bars represents the results for a specific featurization, also indicated by the color. The left bar of each pair shows R^2 when generating 200 members by 2-fold, the right bar when using bootstrapping.

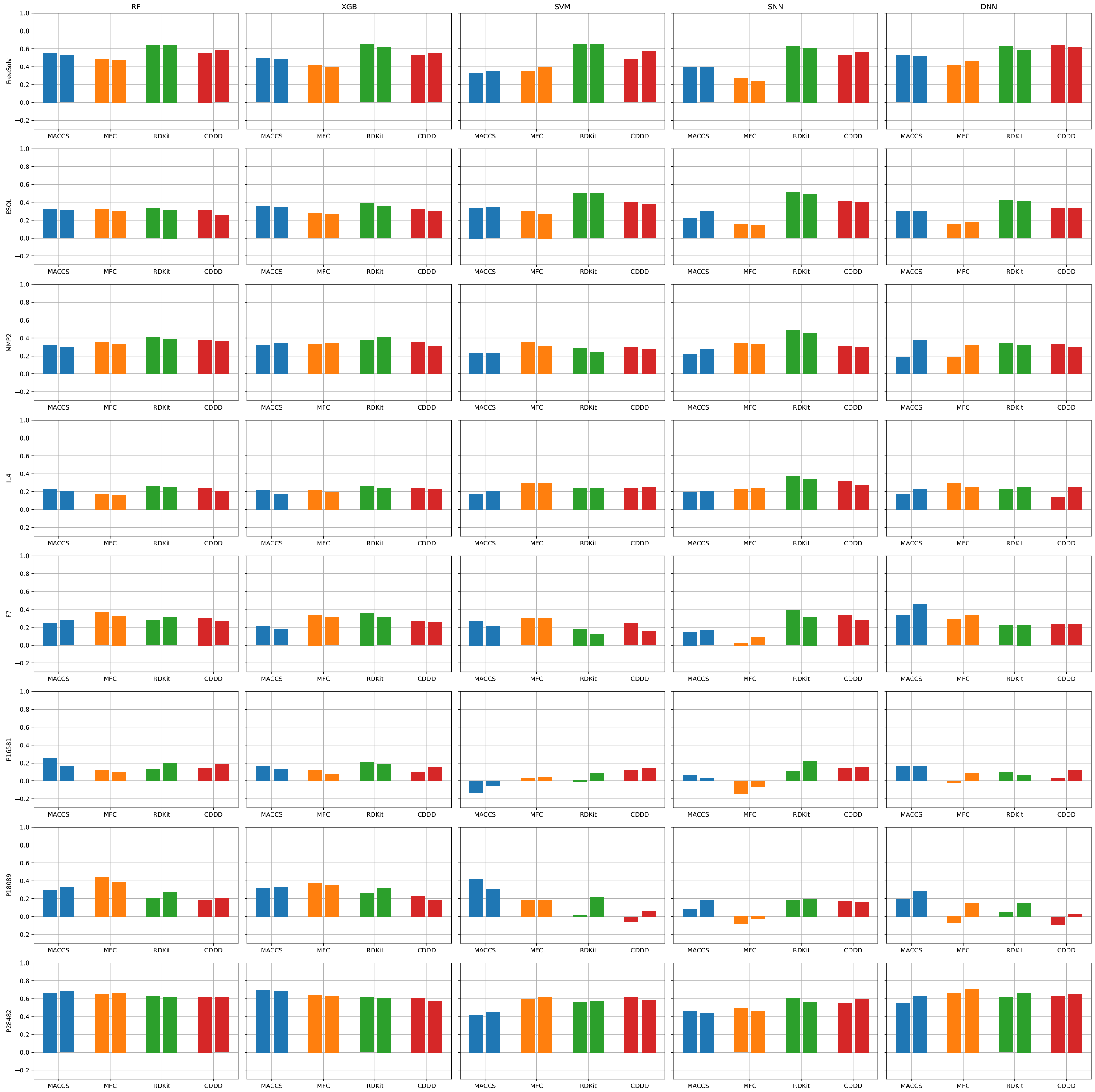

Figure S2.2: Differences in UQ performances between subsampling by 2-fold CV and by bootstrapping for the eight selected datasets. Each row corresponds to the comparisons between results of one dataset, each plot in a row shows the results for a specific modeling technique. Within each plot, a pair of bars represents the results for a specific featurization, also indicated by the color. The left bar of each pair shows ρ when generating 200 members by 2-fold, the right bar when using bootstrapping.

3 Dealing with extreme prediction outliers

Some per-member evaluations resulted in unrealistic predictions. An example of such a case are the predictions of member 40 from the ensemble evaluation of Tetrayhmena, featurized by RDKit descriptors and using the SNN. Assessing the predictive performance from the raw, unfiltered predictions of member 40 is visualized in Fig. S3.1. Especially the SNN was prone to produce such predictions, mostly in combination with RDKit descriptors. Predictions of the SNN showed a noticeable variation in general, where an initialization using different random seeds can distinctively alter the model quality. Furthermore, they were more prone to outliers, which can be extreme in the case of RDKit descriptors. To deal with such predictions, an outlier filter was applied to each set of per-member predictions. Let y^{train}_{max} denote the maximum value of the train output variables and y^{train}_{min} their minimum. Then, the width of the interval covering all train outputs can be defined as $y^{train}_{range} = y^{train}_{max} - y^{train}_{min}$. The filter would then remove test predictions $\hat{y}^{test}$ outside a specified interval wider than y^{train}_{range}, yielding $\hat{\mathbf{y}}^{test}_{filtered}$:

$$\hat{\mathbf{y}}^{test}_{filtered} = \{\hat{y}^{test} \in \hat{\mathbf{y}}^{test} \mid y^{train}_{min} - \delta \cdot y^{train}_{range} < \hat{y}^{test} < y^{train}_{max} + \delta \cdot y^{train}_{range}\}$$

The acceptance level δ was set to 0.5, resulting in a coverage range of twice the train output interval width. In the example, the outlier filter removed three predictions, which were located between 16.90 and 26.99. The result is shown in Fig. S3.2.

An independent variable that often causes extreme outliers for datasets featurized as RDKit descriptors is the shape index Kappa3. In terms of the example, deleting Kappa3 completely and rerunning the evaluation of member 40 also leads to a reasonable predictive performance, as visualized in Fig. S3.3. However, knowing which variables cause outliers in the test set obviously necessitates knowing the test set. Although test data were available for a split as part of a cross-validation, it cannot be guaranteed that future compounds would conform to the remaining descriptors. It was therefore considered more realistic to remove outliers retrospectively, from the predictions, with the filtering approach. The notebook to obtain the figures with additional outlier analysis and an investigation for the influence of Kappa3 can be found in [S4].

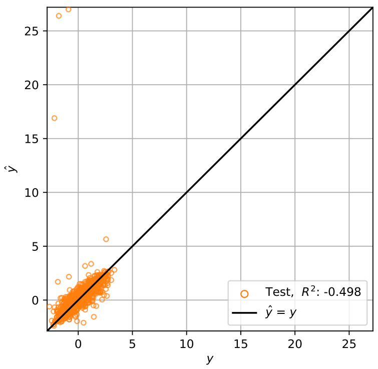

Figure S3.1: Raw predictions.

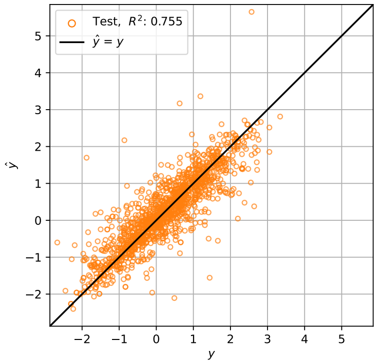

Figure S3.2: Predictions after removing those that are outside the acceptable interval.

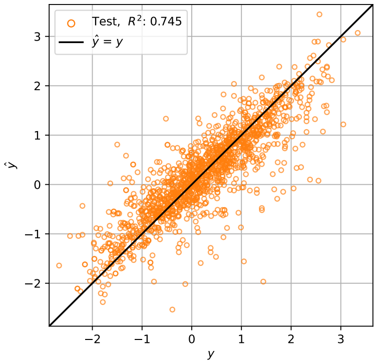

Figure S3.3: Predictions when removing 'Kappa3' from the dataset featurized as RDKit descriptors before machine learning.

4 Full ensemble UQ performances

	RF				XGB				SVM				SNN				DNN			
	MACCS	MFC	RDKit	CDDD	MACCS	MFC	RDKit	CDDD	MACCS	MFC	RDKit	CDDD	MACCS	MFC	RDKit	CDDD	MACCS	MFC	RDKit	CDDD
Tetrahymena	0.40	0.44	0.47	0.43	0.40	0.41	0.52	0.44	0.31	0.39	0.48	0.43	0.32	0.39	0.47	0.42	0.31	0.25	0.44	0.41
FreeSolv	0.56	0.49	0.64	0.56	0.51	0.41	0.66	0.54	0.35	0.35	0.65	0.48	0.39	0.26	0.65	0.54	0.53	0.43	0.64	0.63
ESOL	0.33	0.32	0.34	0.32	0.35	0.28	0.38	0.33	0.35	0.30	0.50	0.39	0.22	0.15	0.52	0.40	0.30	0.16	0.41	0.35
MMP2	0.33	0.36	0.41	0.38	0.33	0.33	0.40	0.36	0.25	0.36	0.31	0.30	0.22	0.35	0.49	0.29	0.17	0.17	0.35	0.32
IL4	0.23	0.19	0.27	0.23	0.22	0.22	0.27	0.24	0.17	0.30	0.24	0.24	0.19	0.24	0.39	0.33	0.17	0.30	0.22	0.14
F7	0.25	0.35	0.27	0.29	0.20	0.34	0.35	0.24	0.30	0.31	0.18	0.24	0.18	0.04	0.39	0.33	0.33	0.30	0.22	0.24
O60674	0.26	0.28	0.33	0.33	0.33	0.30	0.31	0.32	0.22	0.22	0.27	0.15	0.20	0.29	0.20	0.30	0.32	0.34	0.30	0.31
O14965	0.35	0.44	0.41	0.42	0.40	0.41	0.44	0.42	0.24	0.35	0.39	0.37	0.27	0.33	0.34	0.35	0.36	0.41	0.38	0.36
P03372	0.34	0.34	0.37	0.42	0.37	0.37	0.40	0.43	0.23	0.29	0.30	0.33	0.21	0.25	0.36	0.33	0.35	0.32	0.34	0.33
P04150	0.32	0.38	0.34	0.35	0.37	0.36	0.35	0.36	0.25	0.28	0.27	0.29	0.24	0.31	0.26	0.29	0.35	0.37	0.31	0.32
P06401	0.42	0.43	0.42	0.44	0.39	0.41	0.38	0.41	0.37	0.38	0.37	0.40	0.28	0.31	0.30	0.34	0.36	0.39	0.38	0.35
P11229	0.38	0.38	0.42	0.38	0.42	0.39	0.45	0.43	0.42	0.46	0.46	0.40	0.38	0.34	0.28	0.35	0.36	0.44	0.33	0.34
P12931	0.35	0.37	0.36	0.35	0.39	0.36	0.40	0.34	0.21	0.31	0.26	0.31	0.16	0.34	0.26	0.32	0.32	0.41	0.30	0.34
P16581	0.24	0.10	0.16	0.14	0.18	0.10	0.19	0.11	-0.13	0.02	0.00	0.12	0.05	-0.14	0.12	0.10	0.14	-0.04	0.09	0.07
P17252	0.49	0.55	0.52	0.48	0.55	0.52	0.58	0.51	0.24	0.45	0.48	0.49	0.22	0.34	0.34	0.42	0.46	0.52	0.51	0.42
P18089	0.30	0.44	0.21	0.19	0.33	0.39	0.27	0.24	0.42	0.20	0.02	-0.06	0.03	-0.08	0.20	0.20	0.19	-0.02	0.06	-0.06
P19327	0.38	0.44	0.44	0.46	0.43	0.40	0.44	0.50	0.26	0.40	0.36	0.38	0.26	0.27	0.28	0.33	0.33	0.40	0.33	0.29
P21554	0.36	0.37	0.38	0.38	0.39	0.36	0.39	0.40	0.22	0.27	0.30	0.35	0.22	0.30	0.31	0.29	0.42	0.37	0.33	0.30
P24530	0.28	0.32	0.30	0.40	0.28	0.27	0.34	0.40	0.35	0.32	0.29	0.32	0.30	0.23	0.26	0.31	0.25	0.26	0.25	0.27
P25929	0.57	0.51	0.60	0.66	0.56	0.49	0.55	0.62	0.63	0.52	0.51	0.53	0.38	0.40	0.31	0.33	0.56	0.49	0.48	0.47
P28335	0.34	0.37	0.36	0.40	0.38	0.36	0.35	0.35	0.37	0.43	0.38	0.37	0.28	0.34	0.28	0.30	0.34	0.37	0.30	0.28
P28482	0.68	0.65	0.63	0.62	0.69	0.65	0.61	0.59	0.42	0.60	0.56	0.61	0.47	0.51	0.59	0.56	0.55	0.67	0.60	0.63
P35968	0.26	0.32	0.30	0.34	0.31	0.34	0.31	0.31	0.20	0.28	0.28	0.30	0.19	0.36	0.21	0.30	0.32	0.38	0.32	0.34
P41594	0.42	0.38	0.44	0.44	0.43	0.42	0.44	0.42	0.30	0.41	0.33	0.44	0.32	0.35	0.20	0.29	0.43	0.43	0.44	0.44
P42345	0.41	0.40	0.43	0.36	0.44	0.43	0.45	0.43	0.25	0.33	0.38	0.33	0.29	0.38	0.34	0.34	0.42	0.42	0.37	0.30
P47871	0.37	0.42	0.38	0.39	0.40	0.41	0.40	0.40	0.39	0.41	0.29	0.30	0.36	0.30	0.28	0.32	0.35	0.33	0.33	0.35
P49146	0.42	0.41	0.42	0.47	0.40	0.33	0.41	0.40	0.39	0.44	0.28	0.33	0.22	0.21	0.28	0.29	0.35	0.29	0.29	0.26
P61169	0.45	0.46	0.46	0.46	0.42	0.39	0.42	0.43	0.34	0.43	0.42	0.48	0.21	0.29	0.27	0.35	0.42	0.41	0.42	0.43
Q05397	0.29	0.32	0.38	0.32	0.39	0.35	0.37	0.34	0.28	0.30	0.36	0.40	0.19	0.16	0.22	0.20	0.26	0.31	0.30	0.36
Q16602	0.27	0.39	0.28	0.34	0.25	0.32	0.25	0.31	0.24	0.35	0.30	0.26	0.22	0.18	0.23	0.15	0.30	0.26	0.25	0.25
P24941	0.34	0.42	0.38	0.41	0.41	0.42	0.45	0.47	0.26	0.22	0.35	0.34	0.28	0.30	0.32	0.39	0.46	0.45	0.42	0.40
Q92731	0.30	0.32	0.37	0.35	0.32	0.34	0.41	0.37	0.24	0.21	0.30	0.25	0.18	0.28	0.27	0.33	0.33	0.32	0.34	0.31

Figure S4: Overview of the UQ performances (ρ) of the 200-member ensembles, for all datasets and each combination of featurization and modeling technique. Brighter colors correspond to larger values.

5 Obtaining the point of saturation from cumulative member curves

The following example plots for SNN ensembles of P03372 demonstrate how the cumulative member curves were processed to obtain the ensemble size at the supposed saturation. The ”raw” cumulative predictive and UQ performance curves for each featurization when only considering the generation order of the members is visualized in Fig. S5.1. The corresponding notebook for obtaining the raw cumulative member curves can be found in [S5]. Permutating the order of members randomly 200 times, computing a cumulative member curve each time and taking the median performance at each of the 200 values per ensemble size results in Fig. S5.2. The notebook that generates all permutated cumulative member curves can be found in [S6]. Finally, the median cumulative member curves are smoothed by fitting a Michaelis-Menten function through them. The median curves together with the fitted functions and the estimated points of saturation are visualized in Fig. S5.3. In [S7], the notebook is provided that fits the Michaelis-Menten functions and estimates the points of saturation.

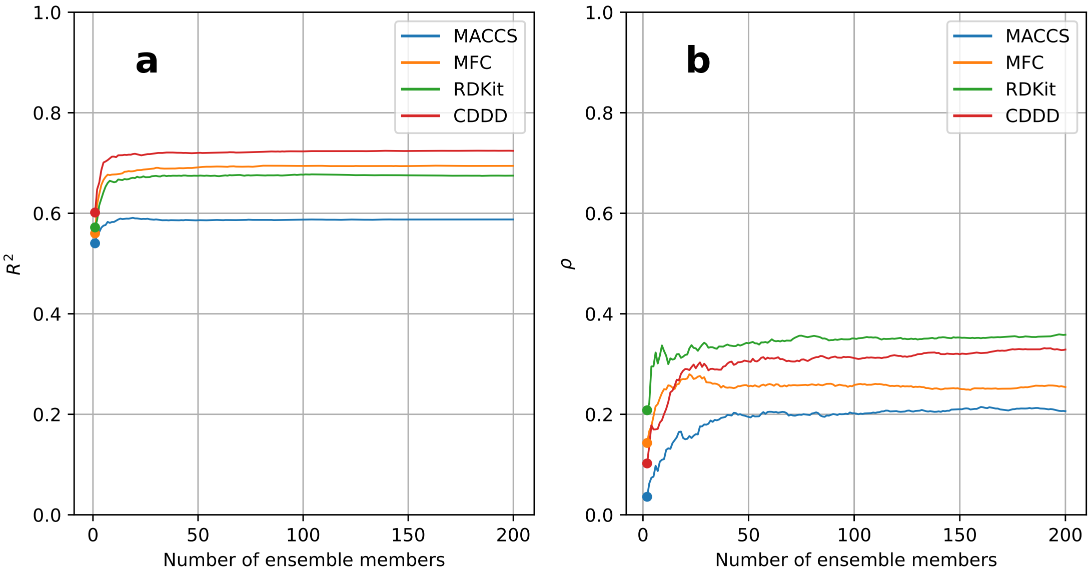

Figure S5.1: Raw cumulative member curves for predictive performance (**a**) and UQ performance (**b**), for all descriptors, modeling P03372, using SNN ensembles.

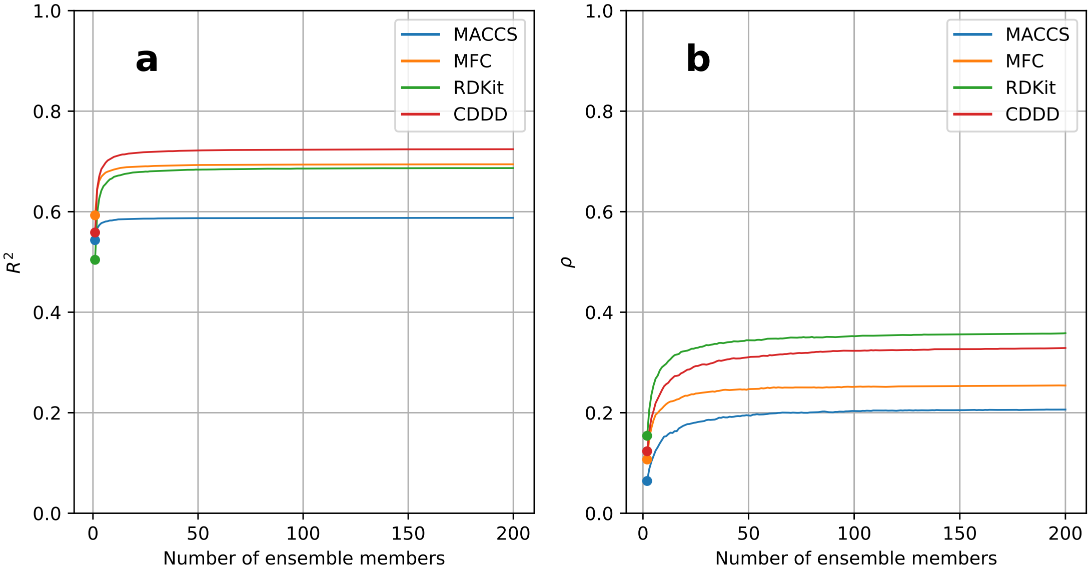

Figure S5.2: Median cumulative member curves for predictive performance (**a**) and UQ performance (**b**), for all descriptors, obtained from 200 permutations of the 200 members per setting.

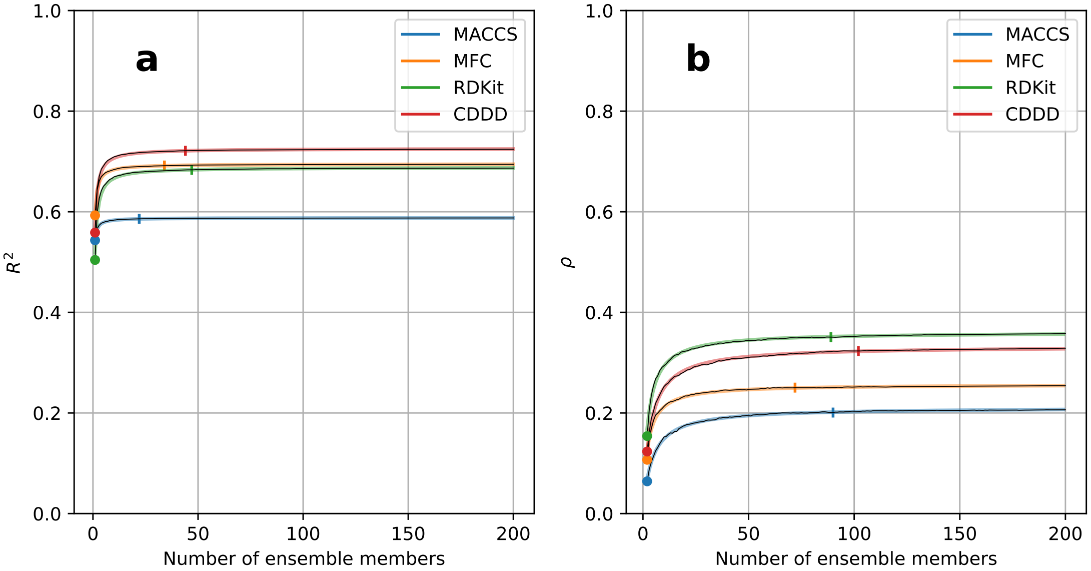

Figure S5.3: Michaelis-Menten functions, fitted to the median cumulative member curves for predictive performance (**a**) and UQ performance (**b**), for all descriptors. The median cumulative member curves are shown as thin black lines. The estimated saturation point for each fitted curve (i.e., the first ensemble size where the corresponding gain in performance falls below 0.0001) is depicted as vertical bar.

6 Ensemble sizes at saturation vs. full ensemble performances

The median ensemble sizes where the saturation was reached was plotted against the performances of the full ensembles, for each dataset, as visualized in Fig. S6. The Spearman's rank correlation coefficient between ensemble sizes and full performances was -0.902 in case of predictive performance and 0.433 in case of UQ performance.

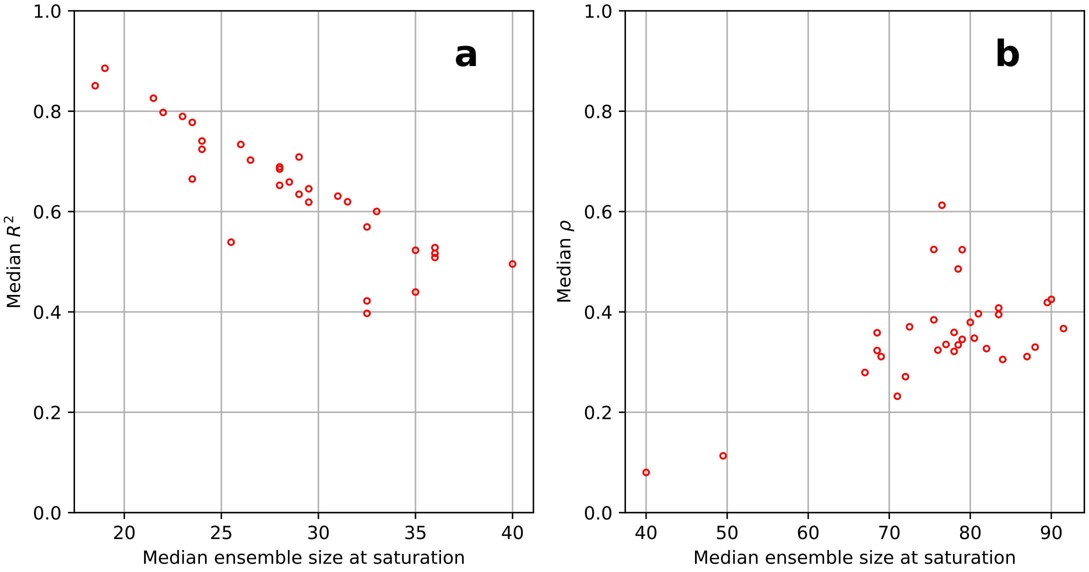

Figure S6: Median ensembles at saturation against full ensemble performance, for predictive performance (**a**) and UQ performance (**b**), for all datasets (32 points in each plot).

7 Single DNN performances

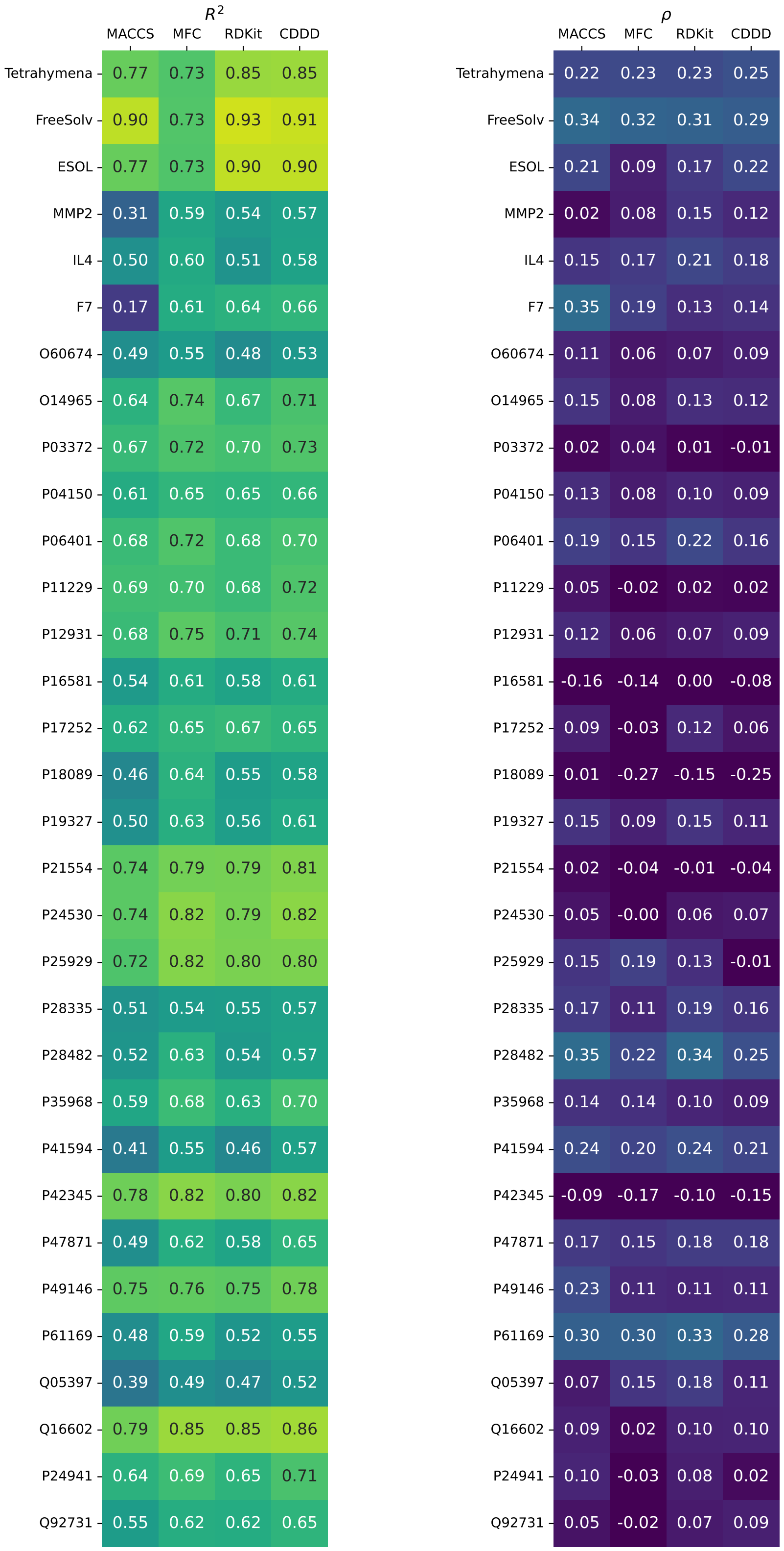

Figure S7: Overview of the predictive performances (R^2) and UQ performances (ρ) of the 10-fold CV single DNN models, for all datasets and each featurization. Brighter colors correspond to larger values.

8 Single RF performances

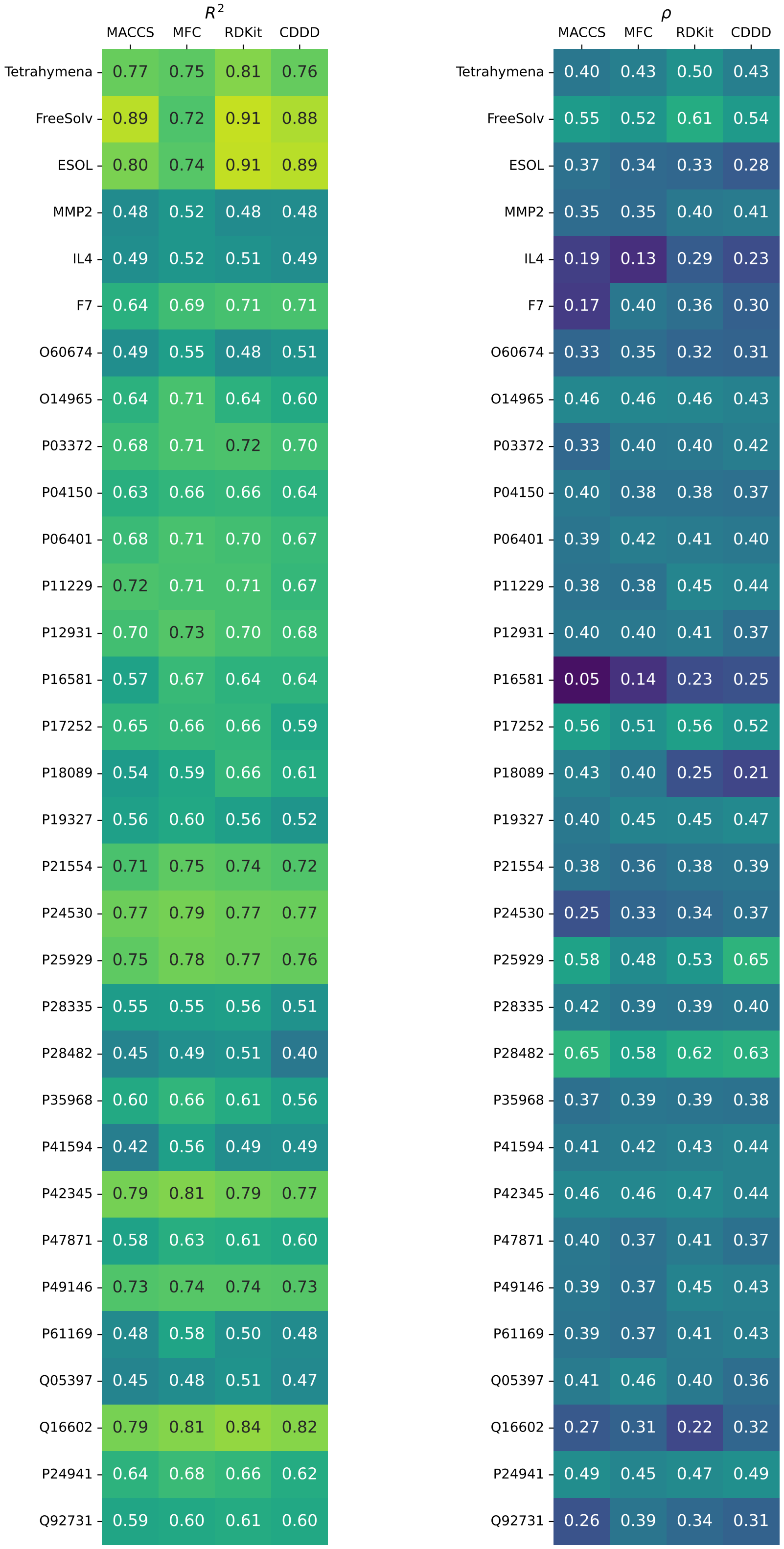

Figure S8: Overview of the predictive performances (R^2) and UQ performances (ρ) of the 10-fold CV single RF models, for all datasets and each featurization. Brighter colors correspond to larger values.

9 Summary of all data sets

Table S1: All data sets used for evaluation. The original number of compounds refers to the number of measurement points in the raw files, the number of compounds after pipeline to the preprocessed files of which descriptor values were computed from.

Name	Category	Original no. compounds	No. compounds after pipeline	Dependent variable	Output unit
Tetrahymena	Non-activity	1571	1448	Inhibitory growth concentration	$pIGC_{50}$
FreeSolv	Non-activity	642	642	Hydration free energy	kcal/mol
ESOL	Non-activity	1144	1117	Aqueous solubility	log mol/L
MMP2	Activity	549	540	Inhibitory concentration	pIC_{50}
IL4	Activity	665	647	Inhibitory concentration	pIC_{50}
F7	Activity	365	357	Inhibitory concentration	pIC_{50}
O60674	Activity	869	866	Inhibitory concentration	pIC_{50}
O14965	Activity	1651	1647	Inhibitory concentration	pIC_{50}
P03372	Activity	908	904	Inhibitory concentration	pIC_{50}
P04150	Activity	1182	1179	Inhibitory concentration	pIC_{50}
P06401	Activity	1233	1231	Inhibitory concentration	pIC_{50}
P11229	Activity	843	683	Inhibitory concentration	pIC_{50}
P12931	Activity	2719	2712	Inhibitory concentration	pIC_{50}
P16581	Activity	184	184	Inhibitory concentration	pIC_{50}
P17252	Activity	580	577	Inhibitory concentration	pIC_{50}
P18089	Activity	137	137	Inhibitory concentration	pIC_{50}
P19327	Activity	1018	880	Inhibitory concentration	pIC_{50}
P21554	Activity	1392	1216	Inhibitory concentration	pIC_{50}
P24530	Activity	1030	955	Inhibitory concentration	pIC_{50}
P25929	Activity	501	467	Inhibitory concentration	pIC_{50}
P28335	Activity	926	896	Inhibitory concentration	pIC_{50}
P28482	Activity	322	319	Inhibitory concentration	pIC_{50}
P35968	Activity	4662	4650	Inhibitory concentration	pIC_{50}
P41594	Activity	1381	1285	Inhibitory concentration	pIC_{50}
P42345	Activity	1337	1337	Inhibitory concentration	pIC_{50}
P47871	Activity	944	599	Inhibitory concentration	pIC_{50}
P49146	Activity	561	486	Inhibitory concentration	pIC_{50}
P61169	Activity	1968	1622	Inhibitory concentration	pIC_{50}
Q05397	Activity	416	415	Inhibitory concentration	pIC_{50}
Q16602	Activity	660	431	Inhibitory concentration	pIC_{50}
P24941	Activity	1130	1129	Inhibitory concentration	pIC_{50}
Q92731	Activity	799	797	Inhibitory concentration	pIC_{50}

10 Python packages with versions

Table S2: Python packages with versions used for this study.

Package	Version
Python itself	3.10.8
Jupyter	3.5.2
Matplotlib	3.6.3
NumPy	1.24.1
pandas	1.5.3
RDKit	2022.09.4
scikit-learn	1.2.0
seaborn	0.12.2
TensorFlow	2.11.0
tqdm	4.64.1
xgboost	1.7.1

11 Additional references

[S1] Bradley Efron. Estimating the Error Rate of a Prediction Rule: Improvement on Cross-Validation. *J. Am. Stat. Assoc.*, 78:316–331, 1983. https://doi.org/10.2307/2288636.

[S2] Wenyu Jiang and Richard Simon. A comparison of bootstrap methods and an adjusted bootstrap approach for estimating the prediction error in microarray classification. *Stat. Med.*, 26:5320–5334, 2007. https://doi.org/10.1002/sim.2968.

[S3] Bradley Efron and Robert Tibshirani. Improvements on Cross-Validation: The .632+ Bootstrap Method. *J. Am. Stat. Assoc.*, 92:548–560, 1997. https://doi.org/10.2307/2965703.

[S4] Thomas-Martin Dutschmann and Lennart Kinzel. Find Explanation for Extreme Outliers. https://git.rz.tu-bs.de/impc/baumannlab/supporting-repository-for-ensemble-publication/-/blob/main/code/notebooks/prediction_outlier_analysis/outlier_inspection.ipynb, Accessed 27 Feb 2023.

[S5] Thomas-Martin Dutschmann and Lennart Kinzel. Check Change in Performance with Increasing Number of Ensemble Members. https://git.rz.tu-bs.de/impc/baumannlab/supporting-repository-for-ensemble-publication/-/blob/main/code/notebooks/cumulative_members/cumulative_members_raw.ipynb, Accessed 27 Feb 2023.

[S6] Thomas-Martin Dutschmann and Lennart Kinzel. Check Change in Performance for the 200 Member Ensembles when Permutating the Member Order and Taking the Median. https://git.rz.tu-bs.de/impc/baumannlab/supporting-repository-for-ensemble-publication/-/blob/main/code/notebooks/cumulative_members/cumulative_members_permutated.ipynb, Accessed 27 Feb 2023.

[S7] Thomas-Martin Dutschmann and Lennart Kinzel. Detect and Visualize the Saturation of each Cumulative Member Curve. https://git.rz.tu-bs.de/impc/baumannlab/supporting-repository-for-ensemble-publication/-/blob/main/code/notebooks/cumulative_members/detect_saturation.ipynb, Accessed 27 Feb 2023.
